# Computational studies on the gas phase reaction of methylenimine (CH_2_NH) with water molecules

**DOI:** 10.1038/s41598-020-67515-3

**Published:** 2020-07-03

**Authors:** Mohamad Akbar Ali

**Affiliations:** Department of Chemistry, College of Science, King Faisal University, 31982, Al-Ahsa, Saudi Arabia

**Keywords:** Atmospheric chemistry, Computational chemistry

## Abstract

In this work, we used quantum chemical methods and chemical kinetic models to answer the question of whether or not formaldehyde (CH_2_O) and ammonia (NH_3_) can be produced from gas phase hydration of methylenimine (CH_2_NH). The potential energy surfaces (PESs) of CH_2_NH + H_2_O → CH_2_O + NH_3_ and CH_2_NH + 2H_2_O → CH_2_O + NH_3_ + H_2_O reactions were computed using CCSD(T)/6–311++G(3d,3pd)//M06-2X/6–311++G(3d,3pd) level. The temperature-and pressure-dependent rate constants were calculated using variational transition state theory (VTST), microcanonical variational transition state theory $(\mu VTST)$ and Rice–Ramsperger–Kassel–Marcus/master equation (RRKM/ME) simulations. The PES along the reaction path forming a weakly bound complex (CH_2_NH⋯H_2_O) was located using VTST and $\mu$VTST, however, the PES along the tight transition state was characterized by VTST with small curvature tunneling (SCT) approach. The results show that the formation of CH_2_NH + H_2_O → CH_2_NH⋯H_2_O is pressure -and temperature-dependent. The calculated atmospheric lifetimes of CH_2_NH⋯H_2_O (~ 8 min) are too short to undergo secondary bimolecular reactions with other atmospheric species. Our results suggest that the formation of CH_2_O and NH_3_ likely to occur in the combustion of biomass burning but the rate of formation CH_2_O and NH_3_ is predicted to be negligible under atmospheric conditions. When a second water molecule is added to the reaction, the results suggest that the rates of formation of CH_2_O and NH_3_ remain negligible.

## Introduction

The alkylamines especially methylamine is emitted to the atmosphere from various sources such as biogenic, oceanic, anthropogenic, animal husbandry, marine emissions, biomass burning (forest vegetation, savannah grass, firewood and agricultural wastes), chemical manufacturing and carbon capture storage (CCS) technologies^1^.

Methylamine (CH_3_NH_2_) is one of the atmospheric precursors of the greenhouse nitrous oxide (N_2_O) gas and HCN and is one of the sources of the formation of NO_x_^2–4^. For example: the synthetic nitrogen-based fuel is one the source of NO_x_ emission^5,6^. Methylamine has also received numerous attention due to its potential role in enhancing particle nucleation and growth and affecting secondary organic aerosol (SOA) formation^7,8^. After emission into the atmosphere, methylamine undergoes conversion reactions in both the gas and aqueous phases. The gas phase reaction of methylamine with hydroxyl radical (CH_3_NH_2_ + OH) is the most important pathway of degradation of methylamine^9,10^. The OH radical initially abstracts the hydrogen from the C–H and N–H groups of the methylamine to generate carbon-centered aminomethyl radical (·CH_2_NH_2_) as a major product and nitrogen-centered methylamine radical (CH_2_N·H) as a minor product^10^. The major detected products from the photooxidation of methylamine are 90% methylenimine (CH_2_NH) and ~ 10% nitrosamine, a carcinogen. The photooxidation reaction of methylamine is given below^1,9,10^

1 $${\text{CH}}_{3} {\text{NH}}_{2} + \cdot {\text{OH}} \to \cdot {\text{CH}}_{2} {\text{NH}}_{2} + {\text{ H}}_{2} {\text{O}}$$

2 $$\cdot {\text{CH}}_{2} {\text{NH}}_{2} + {\text{ O}}_{2} \to \cdot {\text{OOCH}}_{2} {\text{NH}}_{2}$$

3 $$\cdot {\text{OOCH}}_{2} {\text{NH}}_{2} \to {\text{ HOOCH}}_{2} {\text{NH}} \cdot$$

4 $${\text{HOOCH}}_{2} {\text{NH}} \cdot \to {\text{ CH}}_{2} {\text{NH }} + {\text{ HO}}_{2} \cdot$$

Methylenimine has been identified as a potential prebiotic precursor of glycine, and it has been detected spectroscopically in interstellar clouds^11,12^. Methylenimine can also be produced from the decomposition of methyl azides^4^ and 2-azidoacetic acid^13^. Experimental study on the electronic spectrum of CH_2_NH shows the broad absorption of the spectrum of n → π* transition in the region 235–260 nm with the maximum absorption cross-section of ~ 4 × 10^–19^ cm^2^ molecule^−1^ near 250 nm^14^. In another experimental study, Rissanen et al.^10^ used photoionization mass spectrometry to detect the formation of CH_2_NH from CH_2_NH_2_ + O_2_ reaction^10^. Methylenimine is isoelectronic with both formaldehyde and ethylene, therefore it can react in several ways. Various research groups have been predicted the structural and thermochemical properties of reverse reaction i.e., CH_2_O + NH_3_ → CH_2_NH + H_2_O^15–20^. But the atmospheric fate of CH_2_NH with H_2_O is not known with certainty because no direct measurements of its reaction kinetics have been carried out. Recently, we have computed the rate constants for CH_2_O + NH_3_ reaction using ab initio/DFT methods coupled with statistical rate theory^20^. We have proposed the formation of CH_2_O⋯NH_3_, NH_2_CH_2_OH and CH_2_NH from CH_2_O + NH_3_ reaction.

There has been considerable speculation about the atmospheric reaction of methylenimine because this compound is highly reactive, soluble in water, and sticky, thus posing severe experimental challenge^1,21–25^. Some researchers suggested that methylenimine can be either photo-oxidized or can react with water vapor^1,21–25^. Gas-phase theoretical models of the methylenimine chemistry in hot protostellar cores are required to explain the formation of substantially larger organics under interstellar conditions^26^. These molecules can then undergo gas-phase reactions to form more complex species such as amino acids, sugars, and other biologically important molecules surface. For example, the formation of aminomethanol (NH_2_CH_2_OH) from CH_2_NH + H_2_O reaction, which can further react with formic acid (HCOOH) to produce glycine and alanine.

Theoretical studies on the atmospheric degradation of methylenimine initiated by HO and HO_2_ radicals have been performed by various research groups^21–25^. In 2015, Ali and Barker predicted the rate constants for the OH + CH_2_NH reaction using ab initio//DFT methods coupled with variational transition state theory^21^. We suggested that OH + CH_2_NH has similarities with its isoelectronic analogous OH + CH_2_O and OH + CH_2_CH_2_ reactions. The reaction rate constants were predicted in the range of 10^–11^ to 10^–12^ cm^3^ molecule^−1^ s^−1^ under atmospheric condition. In 2016, Vazart et al.^26^ calculated the rate constants for OH + CH_2_NH reaction using DFT and CCSD(T) level. Our group has also predicted the rate constants for HO_2_ + CH_2_NH reaction using ab initio//DFT methods coupled with microcanonical variational transition state theory^22^. Recently, Ali et al.^23,24^ proposed the reaction mechanism of the catalytic effect of a single water molecule on the OH + CH_2_NH, OH + CH_2_O and OH + CH_2_CH_2_ reactions. Ali et al.^23,24^ concluded that a single water molecule has a negative effect on OH + CH_2_NH, OH + CH_2_O and OH + CH_2_CH_2_ reactions. Ali et al. suggested that water-assisted OH + CH_2_NH, OH + CH_2_O and OH + CH_2_CH_2_ reactions cannot accelerate the reaction because the dominated water-assisted process depends parametrically on water concentration. As a result, the overall reaction rate constants are smaller^23,24^.

In earlier studies^1,10,27^, various research groups have been proposed that the hydrolysis of methylenimine will produce CH_2_O + NH_3_, but no clear justification has been made so far. They suggested that, CH_2_NH are water-soluble and will be absorbed by aqueous aerosols in the troposphere. In aqueous solution, it is well known that CH_2_NH undergoes hydrolysis to yield ammonia and formaldehyde. The process of formation of ammonia and formaldehyde is acid-catalyzed and should be relatively rapid in aerosol droplets^1,10,27^. The interest of present work is to address the question of whether the gas phase reaction of CH_2_NH with H_2_O will leads to the formation of formaldehyde and ammonia.

The role of the water molecule as a catalyst in the hydrogen transfer reaction of simple atmospheric and combustion product i.e., ketene (H_2_C=C=O) has been studied by various research groups^28–30^. Nguyen et al.^28^ calculated gas phase pseudo-first-order reaction rate constants for ketene with water molecules at room temperature. The calculated decay rate of ketene was obtained for both the pathways 1.5 × 10^−19^ (pathway A) and 1.5 × 10^−16^ s^−1^ (pathway B). They suggested that the gas phase reaction with ketene and water molecules to form acetic acid is many orders of magnitude slower. They also purposed that room-temperature formation of acetic acid from CH_2_CO + 2H_2_O → NH_3_ + CH_2_O is almost negligible and maybe provide better understanding in the aqueous phase chemistry^28^. The role of two-water reaction with CH_2_NH is also of great interest from the viewpoint of atmospheric and combustion chemistry research. Because CH_2_NH is also isoelectronic analogous to important atmospheric and combustion species *i.e.,* CH_2_O and CH_2_CH_2_.

To the best of our knowledge, there have been no theoretical chemical kinetics investigations on the hydrolysis of CH_2_NH in the gas phase. To investigate the various possibilities of CH_2_NH + H_2_O and CH_2_NH + 2H_2_O reactions, we used ab initio/DFT method for the potential energy surface and advanced kinetic models to predict the temperature- and pressure-dependent rate constants. Finally, concluding remark whether the formation of CH_2_O and NH_3_ is a possible pathway under both atmospheric and combustion conditions are drawn.

## Theoretical methods

### Electronic structure calculations

Geometries of all stationary points were optimized using M06-2X method^31^ in conjunction with Pople 6–311++G(3df,3pd) basis set^32^. The M06-2X has been shown to be reliable for handling noncovalent interactions between molecules and widely used to locate the transition states of atmospheric and combustion reaction systems^20,33–35^. The optimized structure of reactants, complexes, intermediates, and transition states (TSs) are shown in Supporting Information Figure S1 and the cartesian coordinates are given in Supporting Information Table S1. Vibrational frequencies were calculated at M06-2X/6–311++G(3df,3pd) to estimate the zero-point corrections (ZPE) for the reactants, complexes, TSs and products. The frequency calculation also shows that the optimized transition states have single imaginary frequency and reactants, complexes, intermediates and products have all positive vibrational frequencies (see Supporting Information, Table S2). Rotational constants were calculated at the same level to calculate rotational partition functions (see Supporting Information, Table S3). Intrinsic reaction coordinate (IRC) calculations were carried out at M06-2X/6–311++G(3df,3pd) level to confirm the identities of the reactants and products for every transition state. Single point energy calculations were calculated using CCSD(T)/6–311++G(3df,3pd) level of theory^36–39^ (see Supporting Information, Table S4) on M06-2X/6–311++G(3df,3pd) optimized geometries. The combination of CCSD(T)/6–311++G(3df,3pd)//M06-2X/6–311++G(3df,3pd) (designated CC//M06) has been tested by many research groups^20,33–35^ and shown to be reasonably accurate. T1 diagnostic was computed using CCSD(T)/6–311++G(3df,3pd) for all important species was < 0.017, which is acceptable for a single reference wave function^40^. The T1 diagnostic for all the species involved in the reaction are given in Supporting Information Table S5. Gaussian 09 suite of programs was used for all ab initio/density functional theory (DFT) calculations^41^.

### Kinetics

#### High-pressure limit rate constants

The high-pressure limit rate constants for title reaction were calculated using canonical variational transition state theory with small curvature tunneling correction (SCT). The generalized rate constants were calculated by minimizing the transition state dividing surface along the reaction coordinate to get the canonical variational transition state theory (CVT) rate constants, which is given by Eqs. (5) and (6):

5 $$k^{GT} (T,\;s) = \Gamma L^{ \ne } \times \frac{{k_{B} T}}{h}\frac{{Q_{TS}^{ \ne } (T,\;s)(T,\;s)}}{{Q_{R} (T)}}exp\left( { - \frac{{V_{MEP} (s)}}{{k_{B} T}}} \right)$$

6 $$k^{CVT} (T) = \min_{s} k_{{}}^{GT} (T,\;s) = k_{{}}^{GT} (T,\;s^{CVT} (T))$$

where $k^{GT} (T,\;s)$ and $k^{CVT} (T)$ are the rate constants of generalized and canonical variational, transition state theory, respectively, *V*_*MEP*_ is the classical barrier height, Γ is the small curvature tunneling (SCT) correction as implemented in Polyrate^42^, *h* is Planck’s constant, *k*_*B*_ is the Boltzmann constant, and $Q_{TS}^{ \ne }$ and $Q_{R}$ are the total partition functions for the transition state and the reactants, respectively. The rate constants were calculated using dual level direct dynamic approach CVT/SCT with interpolated single point energies (ISPE)^43^. The minimum energy pathway is obtained using direct dynamics for a small range of the reaction path with the mass scaled reaction coordinate ‘*s*’ from − 1.0 to 1.0 bohr by using the Page–McIver integrator with a step size of 0.005 bohr. The SCT transmission coefficients^44^, that include the reaction-path curvature effect on the transmission probability, are based on the centrifugal-dominant small-curvature semiclassical adiabatic ground-state (CD-SCSAG) approximation were computed as discussed in Ref.^44^. The SCT method^44^ was widely used for many atmospheric and combustion reaction systems and provide reasonably accurate value^23,24,33^. All the unimolecular reaction rate constants for non-barrierless reaction were calculated using Polyrate and Gaussrate suites of program^42,45^. The $K_{eq} (T)$ for all the reactants → complexes and complexes → pre-reactive complex were calculated (see Supporting Information Table S6) using THERMO code as implemented in MultiWell Program suite^46–48^. The details procedure of $K_{eq} (T)$ calculations are given in the Supporting Information.

For barrierless reaction, *ktools* program was used to compute rate constants based on variational transition states theory (VTST) and microcanonical variational transition states theory ($\mu$VTST)^46–48^. In the *ktools* program, we supply reactants and the collection of the loose transition states along the reaction coordinate. This can be further achieved by performing a series of constrained optimizations at fixed distances along the reaction path (RP). At each fixed distance, the potential energy was calculated, and optimized geometry was used to obtain the rotational constants and a vibrational analysis was used to obtain the vibrational frequencies of the orthogonal degrees of freedom, after projecting out the reaction coordinate. The zero-point energy from the orthogonal modes, ΔE_z_(*s*), and the electronic energy ΔE_e_(*s*) at each fixed bond distance *s* were used to compute the potential energy with ZPE corrections: V(s) = ΔE_e_(*s*) + ΔE_z_(*s*), where ΔE_e_(*s*) = E_e_(*s*) − E_e_(*s* = 0) and ΔE_z_(*s*) = E_z_(*s*) − E_z_(*s* = 0). The rotational partition functions and vibrational partition functions were computed from rotational constants and the vibrational frequencies, respectively for the orthogonal normal modes evaluated at the fixed distance *s*. Utilizing these parameters, "trial" rate constants or reaction fluxes were computed at each point along the reaction path^20–22^. The point at which the minimum trial rate constant or reaction flux occurs was identified as a variational transition state (VTS). The obtained minimum TS is also compatible to run the master equation (ME) code for pressure- and temperature-dependent rate constants.

#### Pressure-dependent rate constants

The 2-D microcanonical rate constants, $k_{i} (E^{\prime})$ for a specific $J^{\prime}$ were calculated according to Eq. 7:

7 $$k(E^{\prime},\;J^{\prime}) = \frac{{L^{ \ne } }}{h} \times \frac{{G^{ \ne } (E^{\prime} - E_{0}^{T} ,\;J^{\prime})}}{{\rho (E^{\prime},\;J^{\prime})}}$$

where $L^{ \ne }$ is the reaction path degeneracy can be written as the product of a ratio of symmetry factors and a ratio of optical isomers; *h* is Planck’s constant; $G^{ \ne } (E^{\prime} - E_{0} ,\;J^{\prime})$ is the sum of states of the transition state as a function of the active energy $E^{\prime} - E_{0}^{T}$; and $E_{0}^{T}$ is the reaction critical energy, which includes zero-point-energy and centrifugal corrections at temperature T; $\rho (E^{\prime},\;J^{\prime})$ is the 2-D density of states of the reactant molecule. The microcanonical thermally averaged high-pressure limit rate constants were obtained as discussed in Ali et al. work^22^.

For pressure-dependent rate constants, RRKM/ME simulation was used to calculate the rate constants as a function of pressure. The calculated sum of states and density of state from the microcanonical process were used to run master equation (ME) code for pressure-dependent (i.e. falloff curve) rate constants. The vibrational frequencies and the moments of inertia were used to calculate the density of states and the sum of states were based on the Stein–Rabinovitch version of the Beyer–Swineheart algorithm^49,50^. N_2_ gas was used as the bath gas, and the energy transfer model with  $\left\langle {\Delta {\text{E}}} \right\rangle_{{{\text{down}}}}$ = 200 × (T/300)^0.85^ cm^−1^^51^. The Lennard–Jones parameters for a N_2_ gas (σ = 4.74 Å and ε/k_B_ = 82 K) were taken from literature^21^ and the Lennard–Jones parameters for all intermediate species (“Well”) (σ = 4.94 Å and ε/k_B_ = 275 K) were based on the previous study^10^. The initial energy distribution for the simulations was the chemical activation distribution for the combination reaction producing the CH_2_NH⋯H_2_O. The temperature and pressure-dependent rate constants were obtained using MultiWell code. The rate constants were calculated over the temperature range of 200–350 K at N_2_ pressures from 0.01 to 1,000 atm.

## Results and discussion

All the stationary points on the PESs for CH_2_NH + H_2_O reaction were obtained using CC/M06 level. To examine the possible catalytic effect of water molecule on the CH_2_NH + H_2_O reaction, we explored the PES of the addition of one water molecule. After the depiction of reaction pathways are considered (“Reaction pathways and thermodynamics analysis”), for which temperature and pressure-dependent rate constants predicted, the ensuing “Rate constants” details chemical kinetic results of CH_2_NH + H_2_O and CH_2_NH + 2H_2_O reactions and “Atmospheric and combustion implications” describes the atmospheric and combustion implications of CH_2_NH + H_2_O reaction system.

### Reaction pathways and thermodynamics analysis

The hydrolysis of methylenimine forming formaldehyde and ammonia is shown below:

$$H_{2} C = N{-}H\left( {{\text{Methylenimine}}} \right)\, + \,H_{2} O\, \to \,OH - H_{2} C - NH_{2} \left( {{\text{Aminomethanol}}} \right)\, \to \,{\text{CH}}_{{2}} {\text{O}}\, + \,{\text{NH}}_{{3}} .$$

In the first step, hydrogen transfer from water molecule to methylenimine producing aminomethanol. In the second step, hydrogen transfer from aminomethanol followed by removal of ammonia produces formaldehyde (see the scheme in SI).

**Figure 1:**
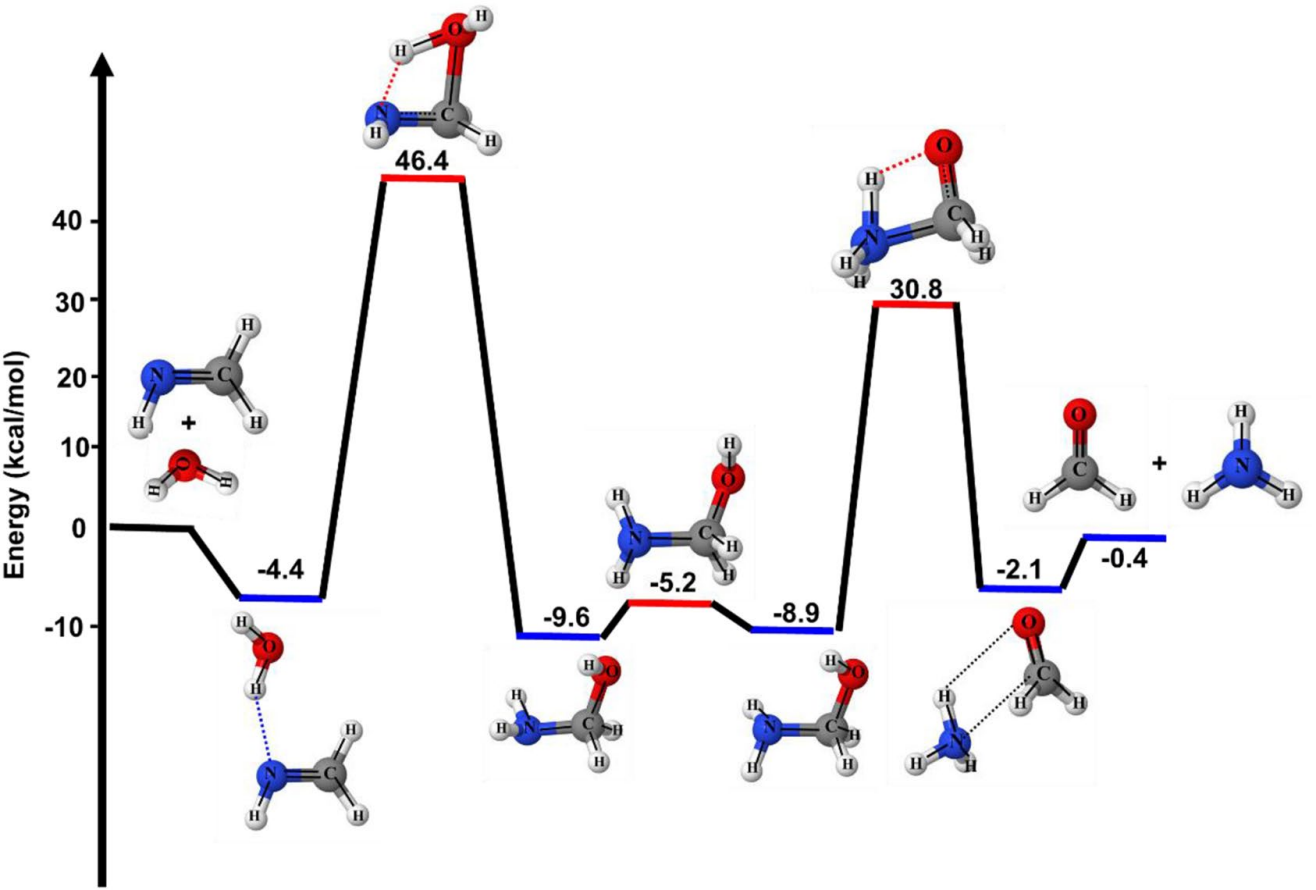
The stationary points on the PES for CH_2_NH + H_2_O reaction were obtained using CCSD(T)/6–311++G(3df,3pd)//M06-2X/6–311++G(3df,3pd). The relative energies include ZPE corrections are relative to CH_2_NH + H_2_O.

The zero-point corrected energies of each stationary point on the PES for the CH_2_NH + H_2_O reaction are tabulated in Table 1 and shown in Fig. 1. Our calculated reaction enthalpy for CH_2_NH + H_2_O → CH_2_O + NH_3_ (− 0.4 kcal/mol) at CC//M06 is in excellent agreement with the experimentally measured value (− 0.3 kcal/mol)^52–54^. Our calculated value is also in excellent agreement with the value obtained by Riffet et al.^15^. Both water ($\mu_{D} = 1.91 {\text{D}}$) and methylenimine ($\mu_{D} = 2.045 {\text{D}}$) are a polar molecule. The partial electric charge on each atom in CH_2_NH and H_2_O are calculated and shown in supporting information Figure S2. Based on charges, we expect they will bind to each other and formed a weakly bound complex i.e., CH_2_NH⋯H_2_O. The calculated binding energies of CH_2_NH⋯H_2_O is 4.4 kcal mol^−1^ lower than the reactants and is also in very good agreement with previous studies^15,16,23^. A hydrogen atom transfer from the H_2_O to methylenimine to form an aminomethanol via four-membered transition state TS1 (Fig. 1). The barrier heights for this transformation is 46.4 kcal/mol, which is 8.2 kcal/mol higher than H_2_O addition to C=O of H_2_C=C=O and 12.3 kcal/mol higher than H_2_O addition to C=C bond of H_2_C=C=O^28^. Once aminomethanol is produced, it can either lose H_2_O molecule to regenerate methylenimine or leads to another hydrogen migration via transition state TS3 to form formaldehyde and ammonia. The barrier heights for the formation of formaldehyde and ammonia is also high (30.8 kcal/mol). This value is also in good agreement with Riffet et al. value^15^.

**Table 1:** Calculated energies (in kcal/mol) for species associated with the reaction of methylenimine with one-water and two-water molecules.

Reaction species	This work^a^	This work^b^	Riffet et al.^c^	Exp.^d^
CH_2_NH + H_2_O	0.0	0.0	0.0	0.0
CH_2_O + NH_3_	− 0.2	− 0.4	− 0.4	− 0.3
CH_2_NH⋯H_2_O	− 6.7	− 4.4	− 3.8	
OH-CH_2_-NH_2_	− 14.9, − 14.2	− 9.6, − 8.9	− 9.6, − 8.8	
CH_2_O⋯NH_3_	− 3.5	− 2.1	− 2.4	
TS1	45.7	46.4	46.8	
TS2	− 10.2	− 5.2	− 5.0	
TS3	28.8	30.8	31.1	
CH_2_NH + 2H_2_O	0.0	0.0	0.0	0.0
H_2_O⋯CH_2_NH⋯H_2_O	− 16.3	− 11.6	− 10.3	
OH-CH_2_-NH_2_⋯H_2_O-1	− 22.6	− 14.9	− 14.8	
OH-CH_2_-NH_2_⋯H_2_O-2	− 23.9	− 15.7	− 14.1	
CH_2_O⋯NH_3_⋯H_2_O	− 11.7	− 7.6	− 6.2	
TS1⋯H_2_O	17.7	20.7	21.3	
TS2⋯H_2_O	− 15.5	− 8.5	− 8.8	
TS3⋯H_2_O	2.9	7.6	7.6	

^a^Calculated at CCSD(T)/6–311++G(3df,3pd)//M06-2X/6–311++G(3df,3pd), ^b^Calculated at CCSD(T)/6–311++G(3df,3pd)//M06-2X/6–311++G(3df,3pd) + ZPE.

^c^Calculated by Riffet et al. work^15^.

^d^From ATcT data^52–54^.

**Figure 2:**
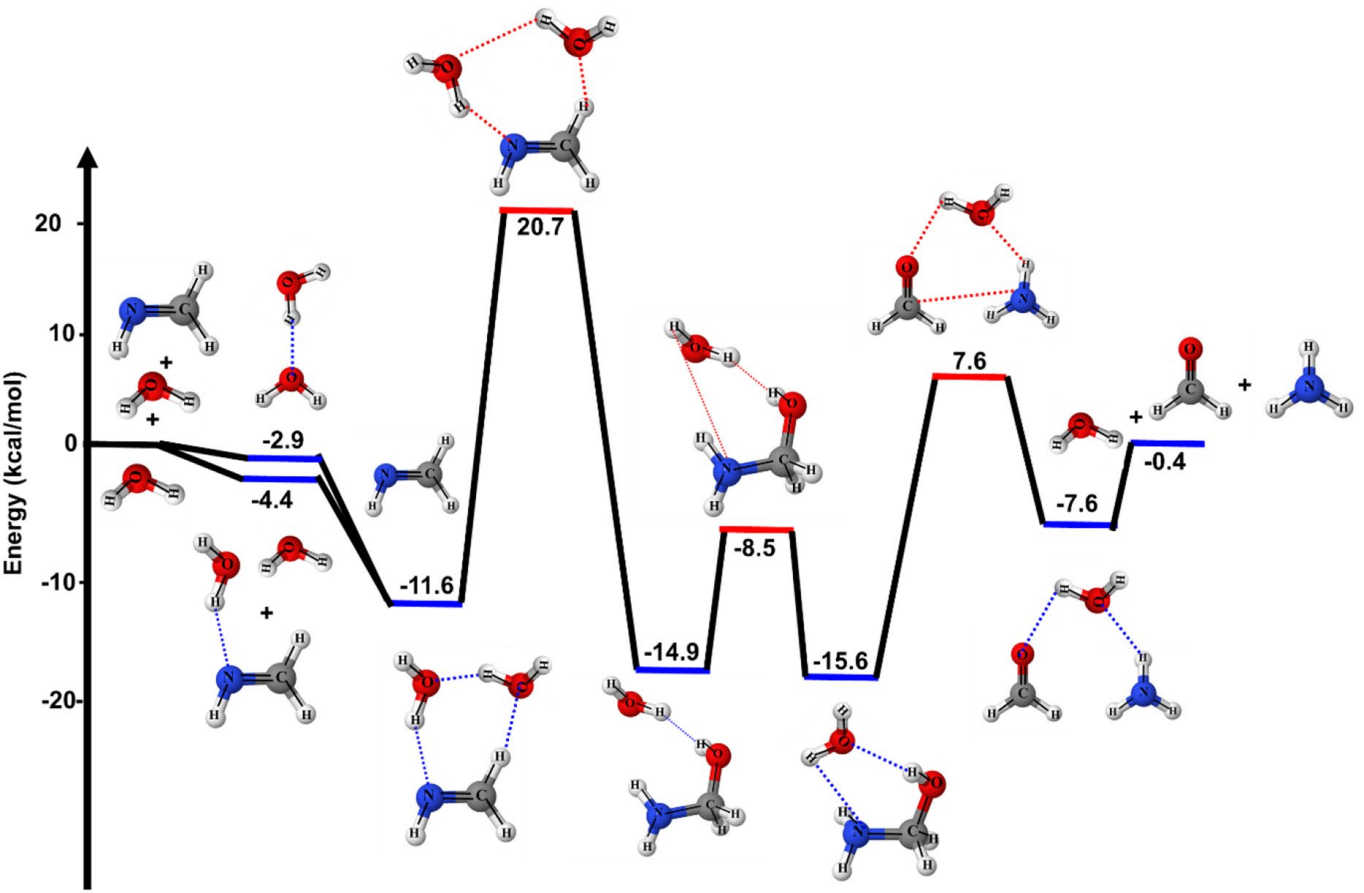
The stationary points on the PES for CH_2_NH + H_2_O + H_2_O reaction were obtained using CCSD(T)/6–311++G(3df,3pd)//M06-2X/6–311++G(3df,3pd). The relative energies include ZPE corrections are relative to CH_2_NH + 2H_2_O.

The zero-point corrected PES for CH_2_NH + 2H_2_O reaction is shown in Fig. 2 and relative energies are tabulated in Table 1. In the presence of two-water molecules, the simultaneous collision of isolated CH_2_NH, H_2_O and H_2_O molecule is very unlikely, therefore, the reaction will occur through the formation of two body complex and then two body complex collides with a third species to form the three-body complex. The calculated binding energies of two body complex i.e., CH_2_NH⋯H_2_O (− 4.4 kcal/mol) are in very good agreement with previously reported (− 3.8, − 4.7 kcal/mol)^15^^,^^23,24^ value . The value for H_2_O⋯H_2_O (− 2.9 kcal/mol) are also in very good agreement with Louie et al.^30^ (− 3.1 kcal/mol) value. As shown in Fig. 2, beginning with the H_2_O + CH_2_NH⋯H_2_O or CH_2_NH + H_2_O⋯H_2_O reactions, a three-body complex H_2_O⋯CH_2_NH⋯H_2_O is formed with the additional water molecule acting as both hydrogen bond acceptor and donor, depending on the approach of the hydrogen atoms in the H_2_O and CH_2_NH molecules. The large binding energies of H_2_O⋯CH_2_NH⋯H_2_O (~ 12 kcal/mol) is due to the combined effects of two O⋯H and one N⋯H hydrogen bonds. The energies of this complex are also in good agreement with Riffet et al. value^15^. It is obvious that water molecule can not only act as reactants but also play an important catalytic role, which makes reaction more thermodynamically feasible (see Fig. 2).

As shown in Fig. 2, the reaction proceeds through a transition state (TS1⋯H_2_O), with a barrier of ~ 21 kcal/mol (with respect CH_2_NH + 2H_2_O) to form an aminomethanol⋯H_2_O. In this process, the six-membered transition state (TS1⋯H_2_O), which has considerably less strain than that of four-membered TS1 of CH_2_NH + H_2_O reaction. The two-water reaction significantly reduced the activation barrier by 25.0 kcal/mol. This result is also consistent with the previous studies on similar reaction system^28,30^. Similar to the one-water reaction, aminomethanol can react catalytically with a single water molecule to either regenerate methylenimine or to form formaldehyde. The barrier heights for the formation of formaldehyde and ammonia is lower (7.6 kcal/mol), and thus should be the dominant channel. As a result, when the aminomethanol is formed, it will rapidly decompose to formaldehyde and ammonia in the presence of water.

Based on the energetics summarized in Table 1, the first barrier heights of a single water reaction is very high (46 kcal/mol). In the case of two water reactions, one water molecule is acting as a catalyst and still does not reduce the barrier heights sufficiently low to allow the hydrolysis of methylenimine to occur readily under typical atmospheric conditions. It has been also reported in the literature that the addition of more water molecules further decreases the barrier heights, but such reactions are not likely to occur in the gas phase^15,28,29^. As discussed in ketene + H_2_O reaction^28^, the gas-phase hydration of ketene by an excess of two water molecules is unlikely to occur at ambient temperatures because the concentration of (H_2_O)_n>2_ clusters in the gas-phase is negligibly small under these conditions. Therefore, the reaction of methylenimine with (H_2_O)_n>2_ is beyond the scope of the present work.

### Chemical kinetics results

#### Rate constants

**Figure 3:**
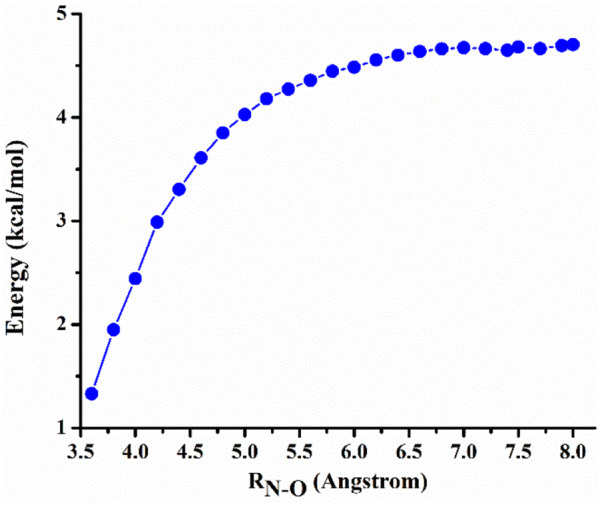
Zero-point corrected potential energy profile for the dissociation of the CH_2_NH⋯H_2_O as functions of R_N-O_ distances.

The CH_2_NH⋯H_2_O complex that plays important roles in the CH_2_NH + H_2_O reaction system are formed via entrance channels that have no intrinsic energy barriers i.e., barrierless. Figure 3 shows the zero-point corrected potential energy for the entrance channel forming CH_2_NH⋯H_2_O.

**Figure 4:**
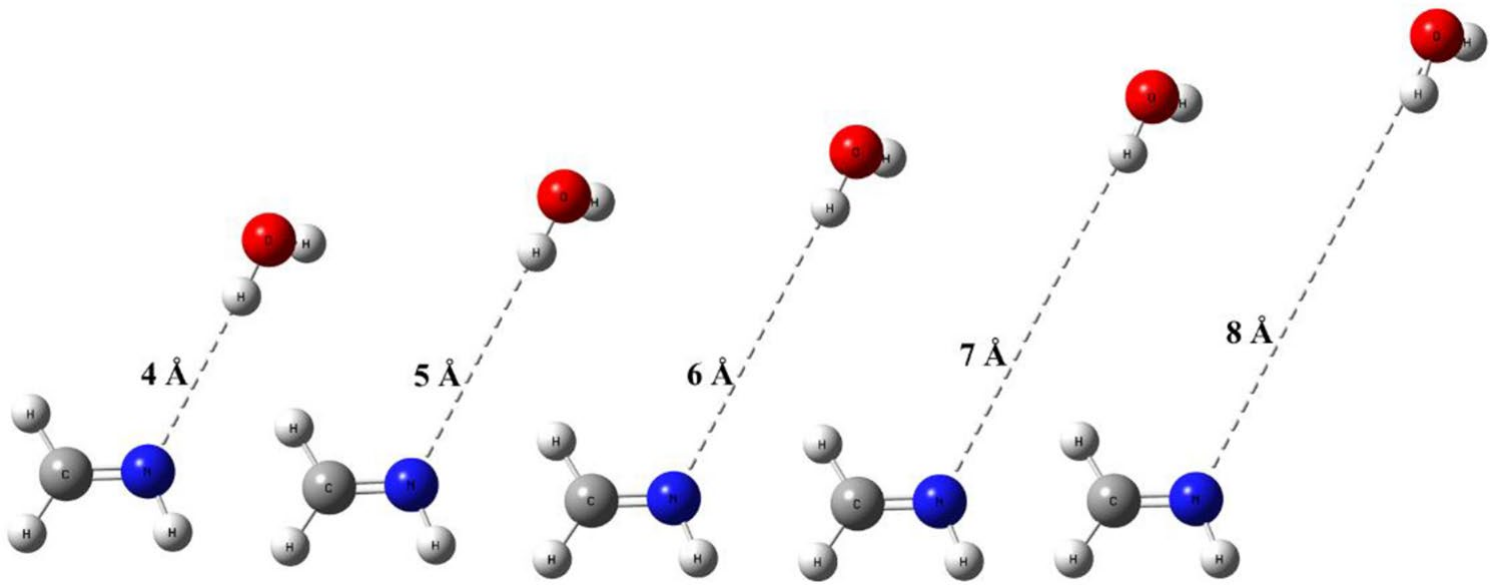
“Trial” TSs of CH_2_NH + H_2_O reaction at several R_N−O_ distances along the reaction pathway forming CH_2_NH⋯H_2_O.

To locate the transition state for the dissociation of CH_2_NH⋯H_2_O, the potential energies (including zero-point energies) were computed in a series of constrained optimizations “trial TS” have one fixed frequency and remaining orthogonal frequency are properly projected as a function of the R_N-O_ bond distance (from 3 to 8 Å). The optimized geometries at some points along the reaction pathways are shown in Fig. 4. All the transition states shown in Fig. 4 have a single imaginary frequency and the normal modes of vibration of these transition states were confirmed to be reliable with the reaction of interest through visualization with GaussView.

At R_N–O_ = 8 Å, the interactions between CH_2_NH and H_2_O are very weak. As the two species approach one another, the potential energy decreases monotonically until it reaches the bottom of the potential well (Fig. 3). The PES decreases to ~ 1 kcal/mol at R_N–O_ = 4 Å and decreased further as CH_2_NH and H_2_O come close to each other. The CVTST "trial" rate constants computed in the temperature range of 200–400 K along the reaction path are shown in Supporting Information Figure S4. At each temperature, the plot shows a single minimum between 5.8 to 6.6 Å. The rate constants for dissociation and association reaction were computed using both canonical and microcanonical approaches and values are tabulated in Supporting Information, Table S7. Our calculation shows that both canonical and microcanonical rate constants for CH_2_NH + H_2_O → CH_2_NH⋯H_2_O reaction is very similar. It is also interesting to know that rate of formation of CH_2_NH⋯H_2_O increases as temperature increases.

**Figure 5:**
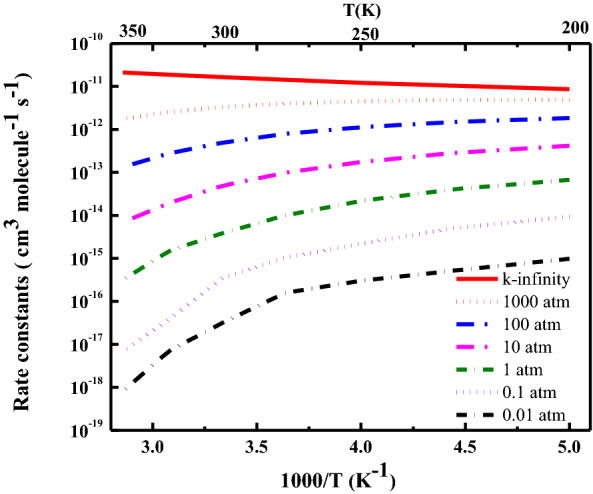
Temperature-and pressure-dependent rate constants for CH_2_NH + H_2_O → CH_2_NH⋯H_2_O.

The sum of states obtained from $\mu$VTST process was used to run the master equation (ME) code for pressure-dependent (*i.e.,* falloff curve) rate constants. Figure 5 shows that microcanonical chemical activation *k* (*E,J*) for CH_2_NH + H_2_O reaction as a function of temperature (200–350 K) and pressure (0.01–1,000 atm). The rate constants are pressure-dependent and show negative temperature-dependence. At lower pressure, rate constants decrease with an increase in temperature and are almost independent of temperature at high-pressure limit. ME simulations show that the formation of CH_2_NH⋯H_2_O is dominant under all the conditions investigated and all other channels i.e., CH_2_NH_2_OH, CH_2_O⋯NH_3_ and CH_2_O + NH_3_ are almost negligible. This result is due to the fact that the barrier heights for the formation of CH_2_NH_2_OH, CH_2_O⋯NH_3_ and CH_2_O + NH_3_ are significantly higher. Our results also suggest that rate constants at high-pressure limit have positive temperature-dependence. This result is similar to the OH + CO and H + O_2_ reaction systems (see examples posted on the MultiWell web site) and the result obtained previously for the CH_2_O + NH_3_ reaction system^20^. We believe that the current microVTST results are accurate to within about a factor of 2.

**Figure 6:**
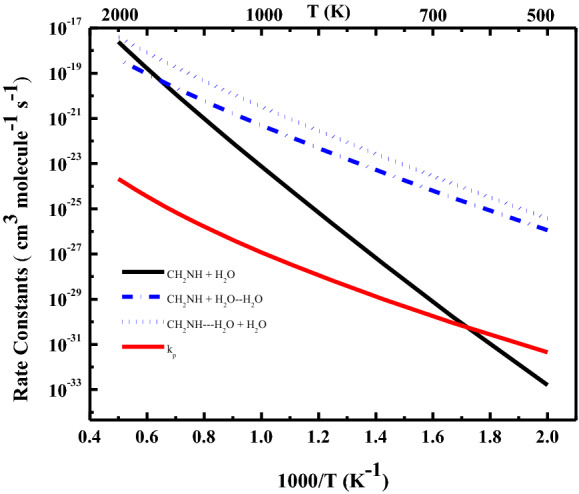
Rate constants for the reaction of methylenimine with one-water and two-water molecules. Dash lines correspond to reaction without water-concentration. It should be noted that these are infinite pressure limit rate constants, which are assumed to be valid from the range T ≥ 300 K, and p ≥ 1 atm.

In some cases, where the rate constant is pressure-independent or depends only weakly on pressure, one does not need to solve ME numerically, and an analytical solution can be obtained. As suggested in earlier studies for a similar reaction system i.e., H_2_O + H_2_C=C=O, the formation of products is almost pressure-independent^28,29^, therefore the rate constants for the reactions of methylenimine with one and two water molecules were calculated based on the high-pressure limit condition as discussed in the method section. The rate constants for the reactions of methylenimine with one water and two water molecules were calculated as a function of temperature are tabulated in Table 2 and shown in Fig. 6.

**Table 2:** Rate constants (cm^3^ molecule^−1^ s^−1^) for CH_2_NH + H_2_O → CH_2_O + NH_3_ and CH_2_NH⋯H_2_O + H_2_O → CH_2_O + NH_3_ + H_2_O (Pathway A) and CH_2_NH + H_2_O⋯H_2_O → CH_2_O + NH_3_ + H_2_O (Pathway B).

Temp (K)	CH_2_NH + H_2_O → CH_2_O + NH_3_	CH_2_NH + H_2_O⋯H_2_O → CH_2_O + NH_3_ + H_2_O	CH_2_NH⋯H_2_O + H_2_O → CH_2_O + NH_3_ + H_2_O	Total effective rate constants (k_P_)
500	1.6 × 10^–33^	1.1 × 10^–26^	3.8 × 10^–26^	4.0 × 10^–32^
600	1.7 × 10^–30^	3.1 × 10^–25^	1.3 × 10^–24^	8.4 × 10^–31^
700	3.6 × 10^–28^	3.9 × 10^–24^	1.9 × 10^–23^	9.1 × 10^–30^
800	2.1 × 10^–26^	2.8 × 10^–23^	1.6 × 10^–22^	6.1 × 10^–29^
900	5.3 × 10^–25^	1.3 × 10^–22^	8.4 × 10^–22^	3.0 × 10^–28^
1,000	7.5 × 10^–24^	4.9 × 10^–22^	3.3 × 10^–21^	1.1 × 10^–27^
1,100	6.7 × 10^–23^	1.5 × 10^–21^	1.1 × 10^–20^	3.6 × 10^–27^
1,200	4.3 × 10^–22^	3.9 × 10^–21^	2.9 × 10^–20^	9.9 × 10^–27^
1,300	2.1 × 10^–21^	8.9 × 10^–21^	7.0 × 10^–20^	2.4 × 10^–26^
1,400	8.5 × 10^–21^	1.9 × 10^–20^	1.5 × 10^–19^	5.5 × 10^–26^
1,500	2.9 × 10^–20^	3.6 × 10^–20^	3.0 × 10^–19^	1.1 × 10^–25^
1,600	8.5 × 10^–20^	6.4 × 10^–20^	5.6 × 10^–19^	2.2 × 10^–25^
1,700	2.2 × 10^–19^	1.1 × 10^–19^	9.8 × 10^–19^	4.2 × 10^–25^
1,800	5.3 × 10^–19^	1.8 × 10^–19^	1.6 × 10^–18^	7.4 × 10^–25^
1900	1.2 × 10^–18^	2.8 × 10^–19^	2.6 × 10^–18^	1.3 × 10^–24^
2000	2.4 × 10^–18^	4.2 × 10^–19^	4.0 × 10^–18^	2.1 × 10^–24^
k = AT^n^ Exp(-Ea/RT)	A = 3.6 × 10^–29^, n = 4.6, Ea/R = 1.9 × 10^4^	A = 9.5 × 10^–31^, n = 4.0, Ea/R = 7.9 × 10^3^	A = 1.2 × 10^–29^, n = 4.1, Ea/R = 8.6 × 10^3^	A = 4.3 × 10^–46^, n = 6.9, Ea/R = 5.41 × 10^3^

As discussed in “Reaction pathways and thermodynamics analysis”, reactions of methylenimine with two-water molecules proceeds through a seven-membered complex, followed by H_2_O addition leading to products. The possibility of the termolecular reaction (i.e*.*, one methylenimine and two free water molecules come together to collide at the same time) is very small under realistic conditions^23,24,28^, therefore, either a N–H hydrogen-bonded CH_2_NH⋯H_2_O complex or a O–H hydrogen-bonded H_2_O⋯H_2_O complex is expected to form first, followed by an attack of the third molecule (H_2_O) to this nascent complex and leading to the following important bimolecular reactions:

**Pathway A**

*Step 0A*

$${\text{CH}}_{{2}} {\text{NH }} + {\text{H}}_{{2}} {\text{O}}\mathop{\longrightarrow}\limits^{{{\mathbf{k}}_{{0{\varvec{A}}}} }}{\text{CH}}_{{2}} {\text{NH}}\cdot\cdot\cdot{\text{H}}_{{2}} {\text{O}}$$

$${\text{CH}}_{{2}} {\text{NH}}\cdot\cdot\cdot{\text{H}}_{{2}} {\text{O}}\mathop{\longrightarrow}\limits^{{{\mathbf{k}}_{{ - 0{\mathbf{A}}}} }}{\text{CH}}_{{2}} {\text{NH }} + {\text{H}}_{{2}} {\text{O}}$$

*Step 1*_*A*_

$${\text{CH}}_{{2}} {\text{NH}}\cdot\cdot\cdot{\text{H}}_{{2}} {\text{O }} + {\text{ H}}_{{2}} {\text{O}}\mathop{\longrightarrow}\limits^{{{\mathbf{k}}_{{1{\mathbf{A}}}} }}{\text{H}}_{{2}} {\text{O}}\cdot\cdot\cdot{\text{CH}}_{{2}} {\text{NH}}\cdot\cdot\cdot{\text{H}}_{{2}} {\text{O}}$$

$${\text{H}}_{{2}} {\text{O}}\cdot\cdot\cdot{\text{CH}}_{{2}} {\text{NH}}\cdot\cdot\cdot{\text{H}}_{{2}} {\text{O}}\mathop{\longrightarrow}\limits^{{{\mathbf{k}}_{{ - 1{\mathbf{A}}}} }}{\text{CH}}_{{2}} {\text{NH}}\cdot\cdot\cdot{\text{H}}_{{2}} {\text{O }} + {\text{ H}}_{{2}} {\text{O}}$$

*Step 2*_*A*_

$${\text{H}}_{{2}} {\text{O}}\cdot\cdot\cdot{\text{CH}}_{{2}} {\text{NH}}\cdot\cdot\cdot{\text{H}}_{{2}} {\text{O}}\mathop{\longrightarrow}\limits^{{{\text{k}}_{{2{\text{A}}}} }}{\text{Products}}$$

**Pathway B**

*Step 0*_*B*_

$${\text{H}}_{{2}} {\text{O }} + {\text{ H}}_{{2}} {\text{O}}\mathop{\longrightarrow}\limits^{{{\mathbf{k}}_{{0{\varvec{B}}}} }}{\text{H}}_{{2}} {\text{O}}\cdot\cdot\cdot{\text{H}}_{{2}} {\text{O}}$$

$${\text{H}}_{{2}} {\text{O}}\cdot\cdot\cdot{\text{H}}_{{2}} {\text{O}}\mathop{\longrightarrow}\limits^{{{\mathbf{k}}_{{ - 0{\mathbf{B}}}} }}{\text{H}}_{{2}} {\text{O }} + {\text{H}}_{{2}} {\text{O}}$$

*Step 1*_*B*_

$${\text{H}}_{{2}} {\text{O}}\cdot\cdot\cdot{\text{H}}_{{2}} {\text{O }} + {\text{ CH}}_{{2}} {\text{NH}}\mathop{\longrightarrow}\limits^{{{\mathbf{k}}_{{1{\mathbf{B}}}} }}{\text{H}}_{{2}} {\text{O}}\cdot\cdot\cdot{\text{CH}}_{{2}} {\text{NH}}\cdot\cdot\cdot{\text{H}}_{{2}} {\text{O}}$$

$${\text{H}}_{{2}} {\text{O}}\cdot\cdot\cdot{\text{CH}}_{{2}} {\text{NH}}\cdot\cdot\cdot{\text{H}}_{{2}} {\text{O}}\mathop{\longrightarrow}\limits^{{{\mathbf{k}}_{{ - 1{\mathbf{B}}}} }}{\text{H}}_{{2}} {\text{O}}\cdot\cdot\cdot{\text{H}}_{{2}} {\text{O }} + {\text{ CH}}_{{2}} {\text{NH}}$$

*Step 2*_*B*_

$${\text{H}}_{{2}} {\text{O}}\cdot\cdot\cdot{\text{CH}}_{{2}} {\text{NH}}\cdot\cdot\cdot{\text{H}}_{{2}} {\text{O}}\mathop{\longrightarrow}\limits^{{{\text{k}}_{{2{\text{B}}}} }}{\text{Products}}$$

As discussed in earlier works^23,24^, locating the TS of backward reaction (i.e*.,* PRC → CH_2_NH + H_2_O⋯H_2_O or PRC → CH_2_NH⋯H_2_O + H_2_O) is more difficult due presence of two or more hydrogen bonds in PRCs, therefore, the equilibrium approach was used to account the presence of forward and backward reactions. This model is reasonably correct when the pre-reactive complex can be stabilized by collisions with other atmospheric species. This approach has been widely used in the literature for the water-assisted reaction and predicted rate constants that are in reasonably good agreement with literature value^23,24^.

As suggested by the reviewer, we have also carried out temperature-and pressure-dependent rate constant calculations for two-water reaction using ME method and results are shown in the supporting information Figure S6. Because of the complexity in locating the TS of loose bonds due to the presence many hydrogens bonded complex in the PRC, we have used Inverse Laplace Transform (ILT) method to account for the barrierless reaction. The ILT parameters used in our calculation are based on our previous work i.e., CH_2_NH + HO_2_ → CH_2_NH⋯HO_2_. Because the first barrier heights is too high (i.e.*,* 32 kcal/mol w.r.t. PRC, see Fig. 2) our ME calculation suggests that the formation of other species (Intermediates and products) are negligibly small in all the temperature and pressure range studied. Based on ME simulation, we have observed that the infinite pressure condition k (T,p), with T ≥ 300 and p ≥ 1 atm for the two-water reaction.

The bimolecular rate constants of pathway A and pathway B were calculated using $k_{A} = K_{eq(A)} \times k_{A}^{CVT}$ and $k_{B} = K_{eq(B)} \times k_{B}^{CVT}$, respectively. The rate constants for these pathways are tabulated in Table 2 and shown in Fig. 6. As shown in the Fig. 6 and tabulated in Table 2, the kinetics of the two-water reaction is more favorable than one-water reaction. This result is due to the fact that the barrier heights of two-water reaction are significantly lower than one-water reaction. As expected, pathway A is more kinetically favorable than pathway B. This result is due to the formation of strong N–H hydrogen-bonded complex (CH_2_NH⋯H_2_O) than weak O–H hydrogen-bonded complex H_2_O⋯H_2_O, which is also consistent with our previous studies^23,24^. It can be seen from Fig. 6, the rate constants for CH_2_NH⋯H_2_O + H_2_O and CH_2_NH + H_2_O⋯H_2_O at < 1000 K are larger than the CH_2_NH + H_2_O reaction and the difference becomes smaller as the temperature increases and is almost similar at the higher temperature (~ 2000 K). It is also clear from this analysis that all reactions involving additional catalytic water are entirely negligible at high temperatures.

The calculated rate constants predict the positive temperature-dependence i.e., rate constants increase with the increase of temperature (see Fig. 6). The tunneling correction for single water and two-water reactions are tabulated in Supporting Information, Table S8. It can be seen from Table S8 (a), the tunneling correction decreases with temperature and at temperature > 1500 K, tunneling correction is almost negligible i.e., $\Gamma$ = 1. In other words, at the combustion condition where the formation of CH_2_O + NH_3_ occurred, tunneling is almost negligible. This result is consistent with previous work on similar reaction system^28^. The tunneling correction of two water reaction is greater than one water reaction at lower temperature and almost negligible at temperature > 1300 K (see Table S8 (b). It should be noted that the *k*(*T*) given in Table 2 has a non-Arrhenius behaviour because of quantum mechanical tunneling effects.

The effective bimolecular rate constants were calculated using with water concentration, which is based on previous works^23,55,56^, therefore the correct expression to calculate the effective rate constants of pathway A and pathway B is given in Eqs. (8) and (9), respectively:

8 $$k_{A}^{eff} = K_{{eq(CH_{2} NH - - H_{2} O)}} \times k_{A} \times [H_{2} O]$$

9 $$k_{B}^{eff} = K_{{eq(H_{2} O - - H_{2} O)}} \times k_{B} \times [H_{2} O]$$

$K_{{eq(CH_{2} NH - - H_{2} O)}}$ and $K_{{eq(H_{2} O - - H_{2} O)}}$ are equilibrium constants for CH_2_NH + H_2_O → CH_2_NH⋯H_2_O and H_2_O + H_2_O → H_2_O⋯H_2_O, reactions, respectively (see Supporting Information, Table S4). The $k_{A}^{{}}$ and $k_{B}^{{}}$ are the bimolecular rate constants of pathway A and pathway B (see Table 2). The [H_2_O] concentration ~ 7.5 × 10^16^ molecule/cm^3^ was used in our calculation is based on the previous study^28^. As shown in Fig. 2, the kinetic scheme CH_2_NH + H_2_O + H_2_O → CH_2_NH⋯H_2_O⋯H_2_O → Products, the rate-determining step is at step 2, which is the same in both pathways. Therefore, the correct equation to calculate the total effective rate constants (*k*_P_) is expressed by Eq. 10

10 $$k_{P}^{{}} = k_{A}^{eff} = k_{B}^{eff}$$

As shown in Fig. 6 and given in Table 2, that effective rate constant at 500 K is five times larger than the one-water reaction. This result suggests that catalytic reaction takes place at a temperature ≤ 500 K. In general, the effective rate constants of the two-water reaction are smaller than the single-water reaction system in the temperature range of 600–2000 K. As a result, H_2_O + CH_2_NH reaction catalyzed by another H_2_O molecule play a minor role for the sink of CH_2_NH in gas phase combustion reaction. Unfortunately, experimental data are not available to validate our predicted rate constants. But we have examined the consistency of present work with earlier theoretical works on similar reaction system^28^. For that purpose, we have calculated the rate constants for termolecular CH_2_NH + H_2_O + H_2_O → CH_2_O + NH_3_ + H_2_O reaction and compared the result with CH_2_CO + H_2_O + H_2_O → CH_3_COOH + H_2_O reaction (see Figure S5)^28^. Our calculated rate constants for CH_2_NH + H_2_O + H_2_O reaction are in good with the value of CH_2_CO + H_2_O + H_2_O reaction in the temperature range of 500–2000 K (see Figure S5).

#### Atmospheric and combustion implications

As reported in previous studies that the methylenimine are likely to hydrolyze easily under atmospheric conditions and hydrolysis of methylenimine will produce NH_3_ + CH_2_O^1,15,27^. It is our interest to know whether or not formaldehyde and ammonia can be produced from the reaction of methylenimine with a single-water and two-water molecules. For this reason, we calculated the pseudo-first-order rate constants for the decay of methylenimine at 1500 K. The water concentration of [H_2_O] ~ 7.5 × 10^16^ molecule cm^−3^ at 50% of relative humidity was used in calculation^28^. The calculated lifetime of methylenimine is ~ 8 min within a factor of 2 errors. The calculated lifetime of methylenimine (~ 8 min.) is also in good agreement with the lifetime of ketene i.e., ~ 5 min. Our calculations suggest that methylenimine can convert to formaldehyde and ammonia only under the combustion condition.

It is important to mention that the reaction of H_2_O + CH_2_NH is still slower than other important reactions for example radical-molecules reaction i.e*.,* CH_2_NH + OH, CH_2_NH + HO_2_ and CH_2_NH + OH (+ H_2_O). Based on our previous works, the CH_2_NH + OH reaction is faster than the H_2_O + CH_2_NH in both atmospheric and combustion conditions^21–23^. The estimated timescales of 8 min are too large to be competitive with the fast-radical reactions i.e.*,* CH_2_NH + OH (+ H_2_O) ~ 1 ns. Therefore, we believe the kinetic of H_2_O + CH_2_NH is even slow in the combustion process compared to radical molecule reactions. Experimental studies are required to validate the formation of CH_2_O and NH_3_ from CH_2_NH + H_2_O reaction.

Now it is important to address the question of whether two-water molecules reaction can produce the formaldehyde and ammonia under atmospheric condition. To understand that, we calculated the pseudo-first-order reaction rate decay of methylenimine at 300 K and at [H_2_O] = 7.1 × 10^17^ molecule/cc at 100% relative humidity. The calculated decay rate is ~ 4.2 × 10^11^ s^−1^ suggest that two-water molecule reaction to form formaldehyde and ammonia is even slower. Therefore, the reaction of two-water molecules on methylenimine cannot produce formaldehyde and ammonia under atmospheric conditions^1^.

To investigate the possibility of the secondary atmospheric reaction with CH_2_NH⋯H_2_O, the atmospheric lifetime of CH_2_NH⋯H_2_O at 225 K and 0.1 atm (i.e., at an altitude of ~ 10–11 km) were calculated and found to be ~ 10 ms. This is a too short lifetime for CH_2_NH⋯H_2_O to undergoes significant bimolecular reactions with other atmospheric species. Thus, although this is an interesting complex, our pressure-dependent rate constants calculation suggests that its formation under the atmospheric condition is unimportant.

Our observation shows that room-temperature formation of formaldehyde and ammonia cannot have come from either of the mechanism of one-water and two-water reaction under atmospheric conditions. Hence, either another mechanism exists or surface reactions similar to that was observed previously are responsible for the formation of formaldehyde and ammonia^28^ The present finding is also consistent with previous studies on the similar type of reactions^28,29^.

## Conclusions

The potential energy surfaces and rate constants for the reaction of methylenimine with one-water and two-water molecules in the gas phase reaction have been calculated using CCSD(T)/M06-2X with 6–311++G(3df,3pd) basis set. For barrierless reactions, rate constants were calculated using canonical and microcanonical variational transition state theory coupled with RRKM/ME simulations and for non-barrierless reactions CVT/SCT approach was used to compute the rate constants. The relative energies of stationary points on the PES are in good agreement with previous values. The barrier heights of one-water reaction are very high. When an additional water molecule is added to the reaction, barrier heights significantly reduced by 25 kcal/mol. Therefore, we can say that an additional water molecule plays a role of a catalysis.

The rate constants for the formation of CH_2_NH⋯H_2_O is both temperature-and pressure-dependent. At the high-pressure limit, the formation of CH_2_NH⋯H_2_O shows weak positive temperature-dependence. Because the lifetime CH_2_NH⋯H_2_O is too short, it is expected to play a negligible role in the atmosphere.

The one-water reaction is to form formaldehyde and ammonia (within a few minutes) is dominant at high temperatures, whereas the two-water reaction becomes the major channel at a lower temperature if step 0 is not included in the calculation. Ignoring step 0 is equivalent to assuming that all the methylenimine is complexed with water, which is not true. Therefore, the correct reaction pathways should have [H_2_O] in the rate constants calculations. In that case, our calculations demonstrate that two-water reaction has the potential to accelerate a gas phase reaction ≤ 500 K, but the rate of formation of formaldehyde and ammonia is predicted to be negligibly slow. This result is also consistent with previous studies on the similar reaction system, i.e., H_2_O + H_2_C=C=O. Experimental studies are required to understand the formation of CH_2_O and NH_3_ from CH_2_NH + H_2_O, and other formation schemes must be explored. Once the laboratory characterization is complete, CH_2_O and NH_3_ will be an ideal target for observational search. Although this is an interesting reaction system, our results demonstrate that CH_2_NH + H_2_O is not important under atmospheric and combustion conditions compared to an important radical molecule reaction. Such results are encouraging, and chemical kinetic mechanism can be useful for the future implementation of hydrolysis of other imine compounds.

## Data availability

All data generated through this study are given in the Supporting Information file. Supporting Information: Tables of optimized geometries, rotational-vibrational parameters, electronic energies, zero-point energies of all the species involved in the CH_2_NH + H_2_O and CH_2_NH + 2H_2_O reactions. Tables of T1 diagnostic for all the species, equilibrium constants, tunneling corrections and rate constants. Figures of optimized structures and comparison of rate constants with other study. Temperature -and pressure-dependent rate constant for one water and two-water reaction. Details discussion on the methodology. Input files for THERMO and KTOOLS are given for replicating the present work.

## Supplementary information

Supplementary file1 (PDF 953 kb)
