# Supplementary material for: Computational studies on the gas phase reaction of methylenimine (CH2NH) with water molecules
Source: Sci Rep. 2020 Jul 3;10:10995. doi: 10.1038/s41598-020-67515-3 (PMC7335075; doi:10.1038/s41598-020-67515-3)
Supplement: Supplementary file 1 — Supplementary file1 (PDF 953 kb) [file 41598_2020_67515_MOESM1_ESM.pdf]

Supplementary Materials

Computational Studies on The Gas Phase Reaction of Methylenimine (CH_2NH) with Water Molecules

Mohamad Akbar Ali [a]

[a]Department of Chemistry, College of Science, King Faisal University, Al-Ahsa 31982, Saudi Arabia..

Corresponding Author E-mail: aamohamada@kfu.edu.sa, akbar256@gmail.com

Table of Contents

Table S1*. Cartesion coordinates (Å) of reactants, complexes, products and transition states obtained using M06-2X/6-311++G(3df,3pd)……………………………………….****Page 3-9***

Table S2*. Harmonic vibrational frequencies (cm^{-1}) of reactants, complexes, transition states and products obatined using M06-2X/6-311++G(3df,3pd)…………………………………………..****Page 10-11***

Table S3: Rotational constants (in GHZ) of reactants, complexes, products and transition states obtained using M06-2X/6-311++G(3df,3pd)………………………………......***Page 12***

Table S4*: Electronic energies + ZPE (Ee + ZPE) (Hartree) of reactants, complexes, products and transition states obtained using CCSD(T)/6-311++G(3df,3pd)//M06-2X/6-311++G(3df,3pd).*
*…………………………………………………………………………………………….****Page 13***

Table S5: *The T1 diagnostic (computed using CCSD(T)/6 311++G(3df,3pd)) for all important species. ……………………………………………… …****Page 14-15***

Table S6**:** Calculated equilibrium constant (cm^3 molecule^{-1}) for the formation of two-body and three-body complex. ……………………………………………………………………………***Page 16***

Table S7*: Capture Rate constants for the CH_2NH +H_2O Entrance Channel using CVTST and μCVTST methods. ……………………………………………………………………………* ***Page 17***

Table S8: Tunneling correction for PRC→ TS→CH_2NH_2OH, and for PRC_w→ TS_w→$CH_2NH_2OH_w$……………………………………………………… ……….***Page 18-19***

Table S9: *Comparison between unprojected and projected Harmonic Vibrational Frequencies of some Trail TS*………………………………………………………….. ***Page 20-21***

Figure S1: Optimized structure of reactants, complex, intermediates, products and transition states. ……………………………………………………………………………………… ***Page 22***

Figure S2: *Calculation Partial Charge on CH2NH and H2O*……………………***Page 23***

Figure S3: Entropy for dissociation of $CH_2NH\cdots H_2O$ as functions of N-O bond distance at 298K. …………………………………………………………………………………………***Page 24***

Figure S4: Trial rate constants for dissociation of $CH_2NH\cdots H_2O$ as functions of N-O bond distance from 200 to 400 K in the interval of 25K. ……………………………………….***Page 25***.

Figure S5*:* Comparison of rate coefficients (a) solid line_Bimolecular (cm^3 $molecule^{-1}$ s^{-1}): CH_2NH+H_2O with CH_2C=O+H_2O (b) Dash line_ Termolecular Reaction (cm^6 $molecule^{-2}$ s^{-1}): CH_2NH+H_2O+H_2O with CH_2C=O+H_2O+H_2O……………………………………………….***Page 26***

Figure.S6 Temperature-and pressure-dependent rate constants for CH_2NH--H_2O+$H_2O\rightarrow$ $H_2O\cdots CH_2NH\cdots H_2O$…………………………………………………………… ***Page 27***

Details of Theoretical Calculations………………………………………………*Page 28-29*
Input Data Files: THERMO and KTOOLS…………………………………………………***Page 30-51***

Table S1: Cartesion coordinates (Å) of reactants, complexes, products and transition states obtained using M06-2X/6-311++G(3df,3pd).

(a) For $CH_2NH+H_2O \rightarrow CH_2O+NH_3$

CH_2NH

C	0.05628700	0.58142600	0.00000000
H	-0.84429800	1.19842500	0.00000000
H	1.00853600	1.10768700	0.00000000
N	0.05628700	-0.67872600	0.00000000
H	-0.89596400	-1.04359100	0.00000000

H_2O

H	0.00000000	0.76099200	-0.46653800
O	0.00000000	0.00000000	0.11663500
H	0.00000000	-0.76099200	-0.46653800

CH_2O

C	0.00000000	-0.52447100	0.00000000
H	-0.93797900	-1.10444600	0.00000000
H	0.93797800	-1.10444800	0.00000000
O	0.00000000	0.66946500	0.00000000

NH_3

N	0.00000000	0.00000000	0.11344200
H	0.00000000	0.93915700	-0.26469800
H	-0.81333400	-0.46957900	-0.26469800
H	0.81333400	-0.46957900	-0.26469800

$CH_2NH\cdots H_2O$

C	-1.25287800	-0.56100700	0.02006900
H	-2.29015400	-0.89421300	-0.02315300
H	-0.47861700	-1.32268900	0.07987300
N	-0.90210100	0.65073100	0.00389800

H	-1.69982300	1.28064600	-0.05584700
H	1.05442400	0.43647600	-0.03364900
O	1.84392400	-0.12431000	-0.07451100
H	2.49475100	0.30519200	0.48116300

$CH_2O\cdots NH_3$

C	0.00000000	1.10058500	0.00000000
H	0.58057500	1.15742200	0.93373000
H	0.58057500	1.15742200	-0.93373000
N	0.86970200	-1.57900100	0.00000000
H	1.21429200	-2.07357000	-0.81381200
H	1.21429200	-2.07357000	0.81381200
O	-1.19226400	0.99458000	0.00000000
H	-0.13953600	-1.67484600	0.00000000

NH_2CH_2OH-1

C	-0.03408900	0.53637700	0.04810600
H	-0.07386700	1.07583700	0.99620800
H	-0.07886200	1.25906300	-0.76295800
N	1.22008500	-0.15859300	-0.01980500
H	1.35295500	-0.78152900	0.76613300
H	1.28647300	-0.70238700	-0.87072100
O	-1.19277200	-0.26399400	-0.11507500
H	-1.28058700	-0.84714400	0.64193400

NH_2CH_2OH-2

C	0.04291000	0.53866700	0.01615000
H	0.03012400	1.19291300	-0.85997400
H	0.08716800	1.16186200	0.91177900
N	-1.12467700	-0.31425600	-0.06016000

H	-1.34225400	-0.72495800	0.83867400
H	-1.93757000	0.18982900	-0.38714400
O	1.20850400	-0.24093200	0.05448200
H	1.10978500	-0.92440300	-0.61497200

TS1

C	0.33637300	0.67471600	0.02082100
H	0.29127600	1.23488300	0.95406000
H	0.16603700	1.27530300	-0.86564000
N	1.07737800	-0.44447500	-0.12130200
H	1.40511100	-0.77001800	0.78306100
H	-0.21668600	-0.86183800	-0.24843300
O	-1.20368200	-0.18360000	-0.08593900
H	-1.57616600	-0.34649600	0.78864700

TS2

C	-0.03598900	0.54626600	0.04282600
H	-0.14847100	1.12461600	0.96255600
H	-0.01039800	1.24136800	-0.79573100
N	1.19209500	-0.24374900	0.11077200
H	0.95820500	-1.20847200	-0.08967400
H	1.85018300	0.04433400	-0.60086700
O	-1.16909100	-0.26590200	-0.15009100
H	-1.42553000	-0.64598700	0.69208700

TS3

C	0.00000000	0.66943500	0.00000000
H	0.27911700	1.24168300	0.89795900
H	0.27911700	1.24168300	-0.89795900
N	0.89042000	-0.62874600	0.00000000

H	1.44033500	-0.79699100	-0.83597100
H	1.44033500	-0.79699100	0.83597100
O	-1.18311200	0.06227600	0.00000000
H	-0.20694900	-1.00297100	0.00000000

(b) For $CH_2NH+2H_2O \rightarrow CH_2O+NH_3 + H_2O$

H_2O ··CH_2NH···H_2O·

C	-1.52975900	-0.87189400	0.03746700
H	-2.38175400	-1.55242300	0.04421700
O	1.73125600	-1.14756800	0.06775700
H	-0.53170900	-1.30147200	0.09658900
N	-1.65196400	0.38359800	-0.04025500
H	-0.02987700	1.28176900	-0.06966800
H	-2.62566400	0.67544900	-0.09585900
H	1.06776200	2.03595100	0.71490100
O	0.92091100	1.51668700	-0.07709700
H	2.42768000	-1.35935200	-0.55449800
H	1.59852600	-0.18669000	-0.00398000

CH_2O···NH_3···H_2O

C	-0.58315427	-1.24592061	0.42316867
H	-0.35971045	-0.69287744	1.34439000
O	-1.25791583	-0.76991882	-0.45190607
H	-0.18256300	-2.26540001	0.34797628
N	1.85949905	-0.24615528	-0.08983096
H	2.77319922	-0.16141329	0.33794934
H	2.00792497	-0.44683146	-1.07155607
H	1.40656449	0.66311418	-0.03797597

O	-0.40787099	1.81553700	0.14221408
H	-0.96498088	1.09537058	-0.18868036
H	-0.83573032	2.63038316	-0.12209195

NH_2CH_2-OH-1···H_2O

C	-1.08263500	-0.10839200	0.42958500
H	-2.15221400	-0.31885800	0.43291000
O	-0.39086500	-1.20014200	-0.19757300
H	-0.71693400	-0.11558800	1.45299600
N	-0.85817500	1.17900000	-0.13829000
H	0.12897500	1.40764800	-0.15956500
H	-1.25066000	1.27554100	-1.06464500
H	2.78672900	-0.15168800	0.44924200
O	2.02089400	0.21426500	0.00565000
H	-0.71982300	-1.30195900	-1.09398200
H	1.38673600	-0.51073000	-0.09105200

NH_2CH_2-OH-2···H_2O

C	1.07830200	-0.11605600	0.38890700
H	0.76029700	-0.10296800	1.43520300
O	0.56828500	-1.21514100	-0.29570200
H	2.16443000	-0.21435100	0.35299500
N	0.59864100	1.13006500	-0.21981800
H	0.88946100	1.93200800	0.32737600
H	0.99288700	1.23000300	-1.14843400
H	-1.27795200	0.66118300	-0.09029300
O	-1.95443500	0.00615500	0.16472300
H	-0.39518400	-1.19129400	-0.20947600
H	-2.70504000	0.14318800	-0.41424700

TS1···H_2O

C	1.19441400	-0.02949200	0.33615400
H	2.10757700	0.40835400	-0.04965100
O	0.00273400	1.39858400	-0.08738000
H	0.97959000	0.17488600	1.37650300
N	0.70329400	-1.13519800	-0.17266700
H	-0.50096700	-1.06175800	-0.01971300
H	1.03163700	-1.34831900	-1.10528900
H	-2.17086800	-0.51037500	0.75329200
O	-1.59738700	-0.38571600	-0.00344900
H	0.10577500	1.66131400	-1.00606800
H	-0.88505700	0.69628900	-0.03069800

TS2···H_2O

C	1.35620600	-0.18403200	-0.06248600
H	2.04935900	-0.33617600	0.77149800
O	0.33409400	-1.15354100	-0.02398300
H	1.89449000	-0.37256700	-0.99018000
N	0.80125500	1.16432300	-0.07620800
H	-0.13950700	1.16353800	0.30320400
H	1.36199000	1.80033300	0.47421300
H	-2.84207100	0.44783200	-0.49342200
O	-2.14750300	0.12468800	0.08219500
H	-0.04111900	-1.17530600	0.86125000
H	-1.52188600	-0.34289900	-0.48388900

TS3···H_2O

C	1.05290200	-0.32965200	0.28431900
H	1.00644200	-0.26125000	1.38279800
O	0.23480200	-1.20771200	-0.27444200
H	2.09865400	-0.41804000	-0.03024900
N	0.59063000	1.10672200	-0.15705800
H	0.98096600	1.85923800	0.40079700
H	0.81970200	1.24709200	-1.13614300
H	-0.51421500	0.98965200	-0.05009600
O	-1.67609200	0.13436600	0.18684800
H	-0.89494500	-0.70142400	-0.05593800
H	-2.41810700	0.10235700	-0.41692900

Table S2: Harmonic Vibrational frequencies (cm^{-1}) of reactants, complexes, products and transition states obtained using M06-2X/6-311++G(3df,3pd).

(a) For CH_2NH+H_2O→CH_2O+NH_3

**CH_2NH**	**H_2O**	**CH_2O**	**NH_3**	**CH2NH···H2O**	**NH2-CH2-OH -1**	**NH2-CH2-OH -2**	**CH_2O··· NH3**	**TS1**	**TS2**	**TS3**
1076.99	1619.24	1219.95	1049.9	118.71	281.53	265.52	45.79	**1900.7*i***	342.8	**1384*i***
1122.4	3889.6	1277.9	1661.9	165.9	396.2	352.5	91.0	433.4	504.3	267.3
1169.7	3990.6	1540.6	1661.9	208.1	488.8	539.1	157.4	471.0	845.7	706.8
1370.4		1884.1	3513.4	209.9	814.7	820.9	185.1	666.2	985.0	789.4
1501.1		2959.7	3637.3	371.3	915.0	974.0	298.1	723.1	1079.1	814.0
1745.0		3030.2	3637.3	618.4	1054.9	1031.3	299.7	764.4	1118.5	1030.9
3068.8		1219.95	1049.9	1081.5	1120.6	1143.5	1077.7	1028.4	1197.4	1198.5
3153.0		1277.9	1661.9	1132.4	1172.8	1227.9	1202.8	1082.9	1295.9	1215.2
3471.7		1540.6	1661.9	1158.3	1372.2	1308.3	1273.5	1254.9	1396.4	1271.9
		1884.1	3513.4	1378.3	1391.0	1391.1	1539.1	1286.0	1449.8	1377.3
		2959.7	3637.3	1503.0	1433.1	1465.5	1658.7	1377.4	1545.9	1482.9
				1642.3	1517.5	1543.1	1663.8	1475.0	1646.3	1543.6
				1749.6	1658.8	1643.9	1865.7	1552.4	3047.6	1566.0
				3076.3	3062.1	3049.6	2988.6	1835.0	3097.1	2226.0
				3177.9	3133.0	3089.3	3059.3	3091.9	3547.4	2970.4
				3496.4	3542.3	3546.7	3503.4	3188.1	3625.3	2992.1
				3719.1	3627.5	3637.1	3625.5	3540.7	3915.7	3493.8

(b) For $CH_2NH+2H_2O \rightarrow CH_2O+NH_3+H_2O$

$H_2O\cdots H_2O$	$H_2O\cdots CH_2NH\cdots H_2O$	OH-CH_2-NH_2-1$\cdots H_2O$	OH-CH_2-NH_2-2$\cdots H_2O$	$CH_2O\cdots NH_3\cdots H_2O$	TS1$\cdots H_2O$	TS2$\cdots H_2O$	TS3$\cdots H_2O$
159.5	64.38	97.09	118.96	82.46	*1326.54i*	*265.66i*	*998.22i*
172.65	78.61	147.41	187.97	110.44	196.47	114.51	164.17
190.29	164.37	184.65	217.51	130.32	300.91	134.73	351.68
207.41	193.83	209.56	262.63	164.49	407.89	175.64	444.89
378.62	211.46	306.79	291.94	188.35	428.53	226.3	449.77
635.48	219.67	409.76	465.34	217.74	516.47	252.03	531.52
1622.83	233.19	450.31	530.71	221.85	584.94	507.27	658.55
1638.71	240.45	517.21	570.4	250.65	601.3	526.69	717.71
3799.08	412.69	643.77	807.27	361.26	692.45	534.27	769.91
3885.07	441.28	823.96	900.76	420.92	733.15	827.54	920.15
	704.08	919.62	989.08	474.64	783.66	1007.28	1044.28
	843.17	1002.62	1035.87	590.77	1014.5	1056.99	1205.99
	1105.89	1117.34	1146.44	1097.76	1111.73	1128.06	1283.77
	1146.34	1190.06	1237.73	1204.66	1231.86	1250.16	1304.87
	1171.71	1366.43	1323.16	1288.11	1297.47	1262.73	1314.47
	1394.03	1396.55	1439.41	1543.27	1408.88	1403.11	1433.75
	1514.09	1429.18	1473.55	1636.3	1470.59	1457.54	1510.91
	1647.34	1512.95	1537.09	1653.89	1498.39	1553.99	1544.16
	1660.69	1627.88	1626.02	1672.73	1562.66	1612.75	1573.83
	1743.46	1664.09	1642.72	1837.83	1638.95	1650.18	1675.78
	3070.29	3081.15	3053.24	3014	1670.93	3015.26	1869.9
	3185.22	3144.56	3095.6	3101.06	1780.36	3099.67	2149.57
	3485.08	3536.98	3528.12	3475.93	3150.72	3528.02	2983.48
	3500.81	3631.83	3556.14	3598.29	3243.92	3624.75	3043.66
	3647.65	3747.92	3614.44	3636.05	3592.16	3817.49	3502.75
	3952.34	3903.03	3772.36	3732.27	3910.38	3877.89	3593.44
	3970.63	3975.67	3967.35	3975.14	3957.8	3972.26	3962.42

Table S3: Rotational constants (GHZ) of reactants, complexes, products and transition states obtained using M06-2X/6-311++G(3df,3pd).

(a) For $CH_2NH+H_2O \rightarrow CH_2O+NH_3$

CH_2NH	H_2O	CH_2O	NH_3	CH2NH··· H2O	NH2-CH2-OH -1	NH2-CH2- OH -2	CH_2O··· NH3	TS1	TS2	TS3
198.84379	830.14341	284.98150	299.22996	34.27515	38.68434	39.17952	34.27515	30.31429	38.51850	33.33499
35.31640	432.95416	39.57973	299.22996	5.01425	9.65297	9.95156	5.01425	10.38226	9.87725	11.24566
29.98993	284.54971	34.75305	189.51096	4.39857	8.66475	8.73185	4.39857	8.58358	8.62782	9.35321

(b) For $CH_2NH+2H_2O \rightarrow CH_2O+NH_3+ H_2O$

$H_2O \cdots H_2O$	$H_2O \cdots CH_2NH \cdots H_2O$	OH-CH_2-NH_2-1···H_2O	OH-CH_2-NH_2-2···H_2O	$CH_2O \cdots NH_3 \cdots H_2O$	TS1···H_2O	TS2···H_2O	TS3···H_2O
214.96040	6.13309	8.84962	8.88416	4.94486	7.77723	9.94246	9.14624
6.51211	3.33696	4.31129	4.60578	4.67846	6.02799	3.88166	5.96131
6.50102	2.18003	3.19148	3.36058	2.63076	3.72609	2.89839	3.98321

Table S4: Electronic energies + ZPE (E_e + ZPE) (Hartee) of reactants, complexes, products and transition states obtained using CCSD(T)/6-311++G(3df,3pd)//M06-2X/6-311++G(3df,3pd).

(a) For CH_2NH+H_2O→CH_2O+NH_3

Species	ZPE	CCSDT	CCSDT+ZPE
CH_2NH	0.040276	-94.474849	-94.4345734
H_2O	0.021641	-76.337436	-76.3157951
CH_2O	0.027139	-114.335966	-114.308827
NH_3	0.034541	-56.476677	-56.4421364
$CH_2NH\cdots H_2O$	0.065563	-170.822989	-170.757426
OH-CH_2NH_2-1	0.070376	-170.836058	-170.765682
OH-CH_2NH_2-2	0.070418	-170.834942	-170.764524
$CH_2O\cdots NH_3$	0.06417	-170.817930	-170.75376
TS1	0.062943	-170.739401	-170.676458
TS2	0.069803	-170.828417	-170.758614
TS3	0.065045	-170.766318	-170.701273

(b) ***For CH_2NH+2H_2O→CH_2O+NH_3+ H_2O***

Species	ZPE	CCSDT	CCSDT+ZPE
H2O$\cdots H_2O$	0.047015	-152.683338	-152.63632
$H_2O\cdots CH_2NH\cdots H_2O$	0.091133	-247.175731	-247.0846
OH-CH_2NH_2-1$\cdots$H2O	0.09577	-247.185695	-247.08993
OH-CH_2NH_2-2$\cdots H_2O$	0.096576	-247.187795	-247.09122
$CH_2O\cdots NH_3\cdots H_2O$	0.0904	-247.168698	-247.0783
TS1$\cdots H_2O$	0.088363	-247.121489	-247.03313
TS2$\cdots H_2O$	0.094811	-247.174473	-247.07966
TS3$\cdots H_2O$	0.091139	-247.145091	-247.05395

Table S5(a): The T1 diagnostic (computed using CCSD(T)/6 311++G(3df,3pd)) for all species involved in CH2NH + H_2O and CH_2NH +2H_2O Reactions.

Species	T1 Diagnostic
CH2NH	0.01228812
H2O	0.00992148
CH2O	0.01542374
NH3	0.00828414
CH2NH⋯H2O	0.01187212
OH-CH2NH2-1	0.01068726
OH-CH2NH2-2	0.01094813
CH2O⋯NH3	0.01373357
TS1	0.01749255
TS2	0.01051416
TS3	0.01475817
H2O⋯CH2NH⋯H2O	0.01178066
OH-CH2NH2-1⋯H2O	0.01081137
OH-CH2NH2-2⋯H2O	0.0110812
CH2O⋯NH3⋯H2O	0.01345468
TS1⋯H2O	0.01635814
TS2⋯H2O	0.01055299
TS3⋯H2O	0.01310301

Table S5(b): The T1 diagnostic (computed using CCSD(T)/6 311++G(3df,3pd)) for all "Trial" TSs and Complex

Species	T1-Diagonistic
Complex	0.01187212
4	0.01152359
4.2	0.0115008
4.4	0.01148309
4.6	0.01146862
4.8	0.01145654
5	0.0114467
5.2	0.01143887
5.4	0.01143272
5.6	0.0114278
5.8	0.01142384
6	0.0114206
6.2	0.01141789
6.4	0.01141567
6.6	0.01141383
6.8	0.01141228
7	0.01141228
7.2	0.01140985
7.4	0.0114089
7.6	0.01140736
7.8	0.01140671
8	0.01542374

Table S6: Calculated equilibrium constant (cm^3 $molecule^{-1}$) for the formation of two-body and three-body complex.

Temp	Ke ($H_2O\cdots H_2O$)	Ke($CH_2NH\cdots H_2O$)	Ke($H_2O\cdots H_2O$+CH_2NH)	Ke($CH_2NH\cdots H_2O$ +H_2O)
200	3.4E-21	1.0E-20	8.3E-18	3.6E-18
225	1.4E-21	2.7E-21	7.0E-19	4.4E-19
250	6.9E-22	9.9E-22	9.7E-20	8.3E-20
275	4.0E-22	4.3E-22	2.0E-20	2.2E-20
300	2.6E-22	2.2E-22	5.2E-21	7.1E-21
325	1.8E-22	1.3E-22	1.7E-21	2.8E-21
350	1.3E-22	8.0E-23	6.7E-22	1.3E-21
375	1.0E-22	5.4E-23	3.0E-22	6.4E-22
400	8.3E-23	3.9E-23	1.5E-22	3.6E-22
500	4.8E-23	1.6E-23	1.9E-23	6.6E-23
600	3.6E-23	9.1E-24	5.4E-24	2.3E-23
700	3.1E-23	6.7E-24	2.3E-24	1.2E-23
800	3.0E-23	5.5E-24	1.3E-24	7.2E-24
900	2.9E-23	5.0E-24	8.3E-25	5.2E-24
1000	3.0E-23	4.7E-24	6.1E-25	4.1E-24
1100	3.2E-23	4.7E-24	4.9E-25	3.5E-24
1200	3.4E-23	4.7E-24	4.1E-25	3.1E-24
1300	3.7E-23	4.8E-24	3.6E-25	2.9E-24
1400	4.0E-23	5.0E-24	3.3E-25	2.7E-24
1500	4.3E-23	5.2E-24	3.1E-25	2.6E-24
1600	4.7E-23	5.5E-24	3.0E-25	2.6E-24
1700	5.1E-23	5.9E-24	2.9E-25	2.6E-24
1800	5.5E-23	6.2E-24	2.9E-25	2.6E-24
1900	6.0E-23	6.7E-24	2.9E-25	2.7E-24
2000	6.6E-23	7.1E-24	2.9E-25	2.7E-24

Table S7: Capture Rate constants for the CH_2NH +H_2O Entrance Channel using CVTST and μCVTST methods.

T (K)	Canonical Approach		Microcanonical Approach	
	Dissociation (s^{-1})	Association (cm^3 $molecule^{-1}$ s^{-1})	Dissociation (s^{-1})	Association (cm^3 $molecule^{-1}$ s^{-1})
200	8.72E+08	8.78E-12	8.57E+08	8.62E-12
225	3.97E+09	1.09E-11	3.77E+09	1.04E-11
250	1.35E+10	1.33E-11	1.24E+10	1.22E-11
275	3.71E+10	1.61E-11	3.29E+10	1.43E-11
300	8.42E+10	1.87E-11	7.39E+10	1.64E-11
325	1.62E+11	2.07E-11	1.47E+11	1.87E-11
350	2.85E+11	2.30E-11	2.63E+11	2.12E-11
375	4.67E+11	2.54E-11	4.36E+11	2.37E-11
400	7.20E+11	2.81E-11	6.77E+11	2.64E-11

Table S8: (a) Tunneling correction for PRC→ TS→CH_2NH_2OH .

Temp	Tunneling
500	5.5
600	2.6
700	2.0
800	1.6
900	1.5
1000	1.4
1100	1.3
1200	1.2
1300	1.2
1400	1.1
1500	1.1
1600	1.1
1700	1.1
1800	1.1
1900	1.0
2000	1.0

Table S8: (b) Tunneling correction for PRC_w→ TS_w→$CH_2NH_2OH_w$

Temp	Tunneling
500	2.9
600	1.9
700	1.6
800	1.4
900	1.3

1000	1.2
1100	1.2
1200	1.2
1300	1.1
1400	1.1
1500	1.1
1600	1.1
1700	1.1
1800	1.1
1900	1.1
2000	1.1

Table S9 (a): Unprojected Harmonic Vibrational Frequencies of some of the Trail TS.

			Un-Projected		
**Bond Length (Å)**	**4**	**5**	**6**	**7**	**8**
	-65.08	-32.34	-18.82	-10.71	-7.23
	58.21	58.31	50.27	43.35	44.15
	65.12	84.28	88.18	85.96	84.16
	126.6	152.17	108.95	138.85	94.21
	203.56	181.89	173.37	169.01	172.24
	378.56	241.75	211.08	193.15	200.74
	1084.84	1083.48	1083.23	1083.07	1083.3
	1120.57	1123.65	1123.82	1123.15	1123.07
	1168.74	1171.75	1172.89	1173.14	1173.03
	1377.86	1375.7	1375.02	1374.6	1374.89
	1503.01	1504.32	1504.86	1504.95	1504.91
	1636.83	1630.82	1627.51	1628.38	1628.4
	1754.74	1754.3	1753.75	1753.32	1753.81
	3063.24	3066.66	3062.3	3059.52	3058.7
	3158.03	3165.15	3167.36	3167.87	3167.44
	3487.62	3470.91	3465.21	3463.93	3462.77
	3867.21	3889.38	3898.3	3901.61	3899.33
	3974.25	3991.59	3997.42	4001.27	3999.56

Table S9 (b): Projected Harmonic Vibrational Frequencies of some of the Trail TS

		Projected			
Bond Length (Å)	***4***	***5***	***6***	***7***	***8***
	57.3	55.8	48.5	42.8	44.1
	63.0	81.8	82.6	75.3	65.8
	126.5	151.7	108.4	138.6	94.2
	203.1	181.9	173.4	168.5	172.2
	377.9	241.5	211.1	193.1	199.3
	1084.4	1082.6	1082.5	1048.2	993.4
	1120.5	1122.0	1107.7	1084.6	1083.3
	1168.7	1171.7	1172.1	1170.2	1169.0
	1306.7	1303.7	1308.4	1307.5	1342.5
	1494.9	1496.4	1499.5	1501.1	1504.8
	1625.4	1616.3	1608.1	1622.4	1602.6
	1723.8	1722.2	1724.7	1723.0	1734.2
	3063.2	3066.5	3061.8	3059.0	3055.7
	3158.0	3165.1	3167.3	3167.9	3166.1
	3465.0	3447.0	3434.4	3412.9	3410.1
	3835.4	3858.1	3868.8	3832.3	3863.2
	3952.6	3969.6	3976.9	3953.4	3981.3

(a) For $CH_2NH+H_2O \rightarrow CH_2O+NH_3$

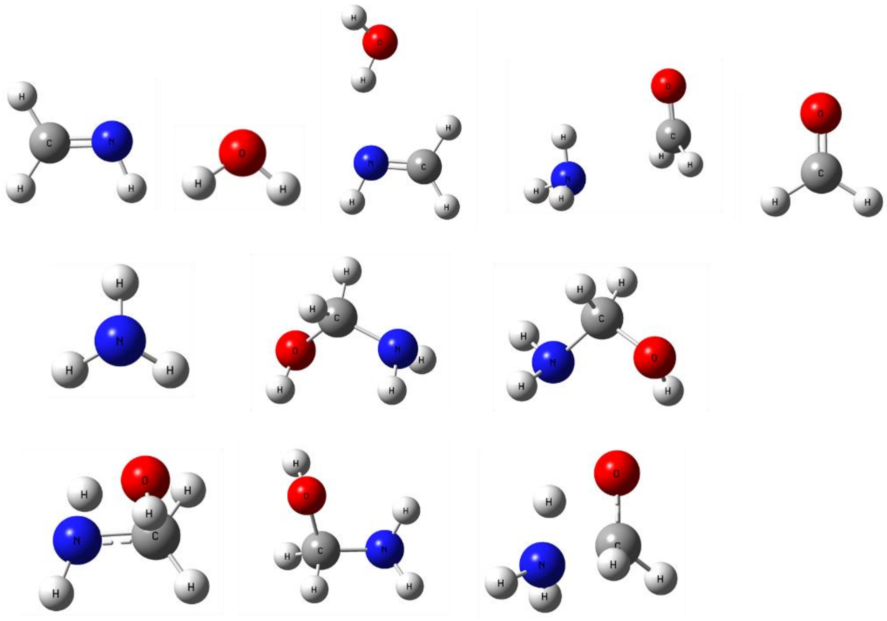

(b) For $CH_2NH+2H_2O \rightarrow CH_2O+NH_3 +H_2O$

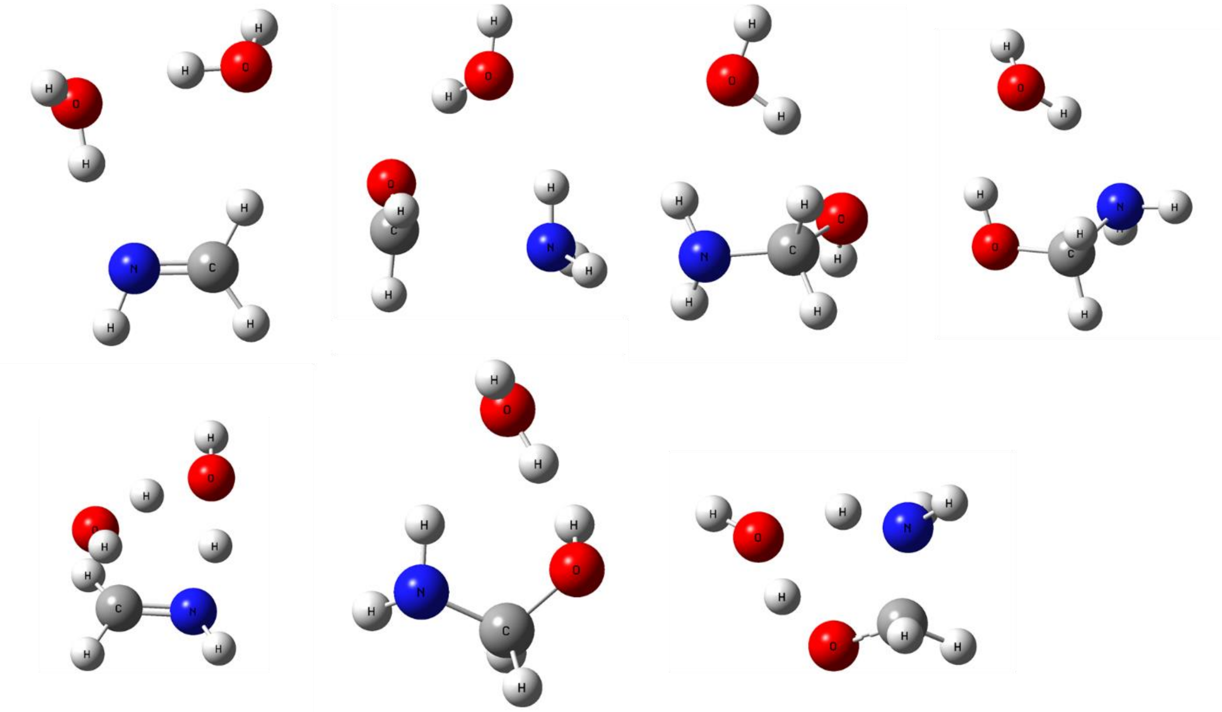

Figure S1: Optimized structure of reactants, complex, intermediates, products and transition states.

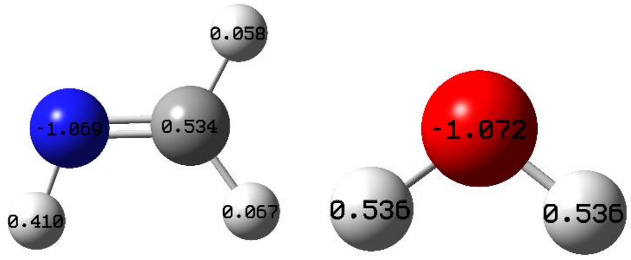

Figure S2: Calculated partial electric charge on each atom in CH_2NH and H_2O.

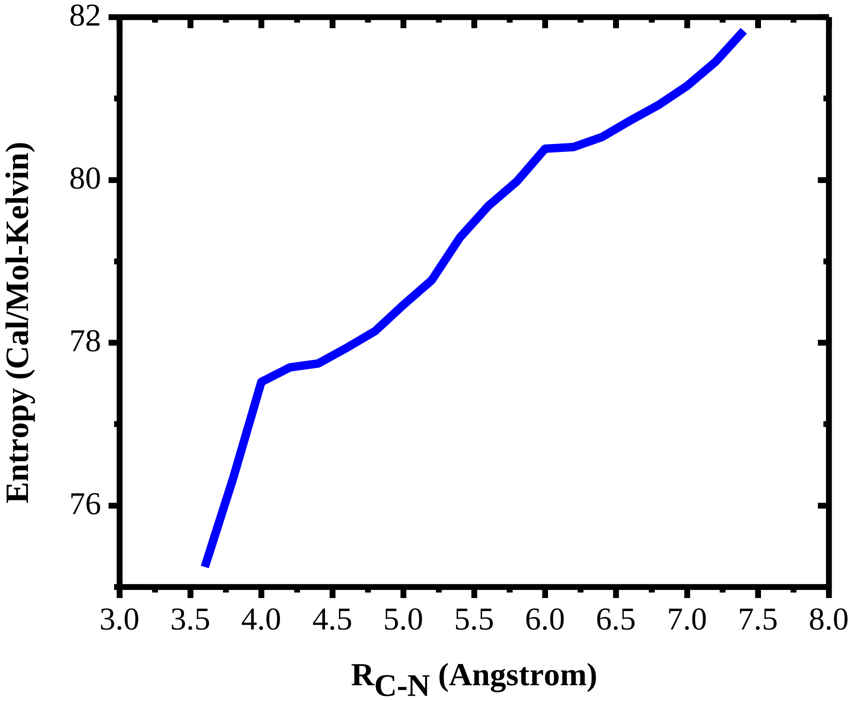

Figure S3: Entropy for dissociation of $CH_2NH \cdots H_2O$ as functions of N-O bond distance at 298K.

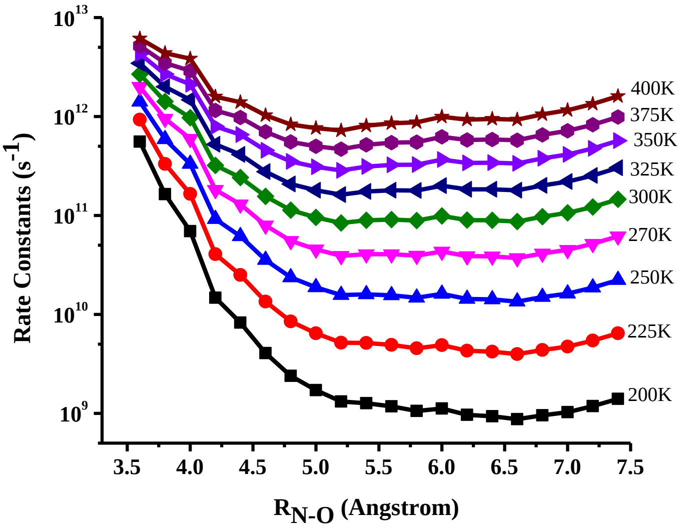

Figure S4: Trial rate constants for dissociation of $CH_2NH\cdots H_2O$ as functions of N-O bond distance from 200 to 400 K in the interval of 25K.

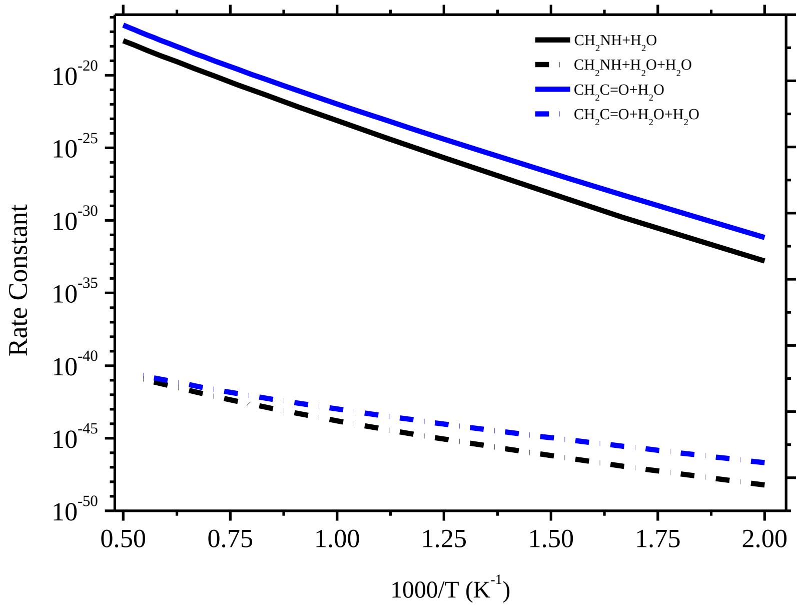

Figure S5: Comparison of rate coefficients (a) solid line_Bimolecular (cm^3 $molecule^{-1}$ s^{-1}): CH_2NH+H_2O with $CH_2C{=}O+H_2O$ (b) Dash line_ Termolecular Reaction (cm^6 $molecule^{-2}$ s^{-1}): $CH_2NH+H_2O+H_2O$ with $CH_2C{=}O+H_2O+H_2O$.

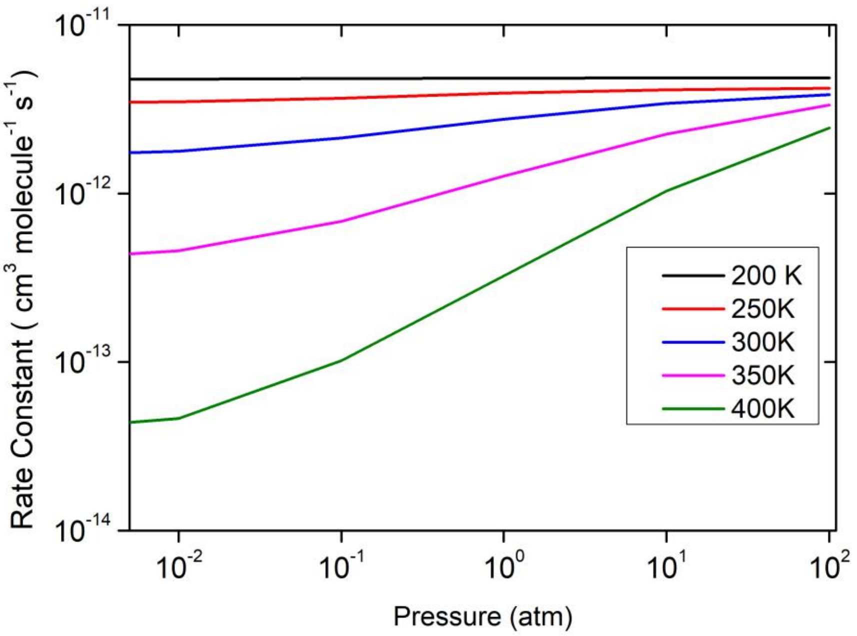

Figure.S6 Temperature-and pressure-dependent rate constants for CH_2NH--H_2O+H_2O→ H_2O···CH_2NH···H_2O.

Details of Theoretical Calculations:

The rate constants were calculated using dual dynamic level with the interpolated single-point energies (ISPE).[40] The minimum energy pathway is obtained using direct dynamics approach for a small range of the reaction path with the mass scaled reaction coordinate from -1.5 to 1.5 Bohr by using the Page–McIver integrator with a step size of 0.05 Bohr. The bimolecular rate constants for one-water reaction were calculated using Eq.S1:

$$k(T)_{\infty} = k^{CVT}(T) \times K_{eq}(T) \qquad \text{(S1)}$$

The equilibrium constants $K_{eq}(T)$ were calculated by EqS2:

$$K_{eq}(T) = \frac{Q_{CH_2NH--H_2O}}{Q_{CH_2NH} \times Q_{H_2O}} \times \exp\left(-\frac{E_0^{CH_2NH--H_2O} - E_0^{CH_2NH} - E_0^{H_2O}}{k_B T} \right).$$

(S2)

Where $Q_{CH_2NH--H_2O}$ Q_{CH_2NH} and Q_{H_2O} are total partition functions for pre-reactive complex, methylenimine and water, and E_0 is the zero-point corrected energies of complex and reactants.

The bimolecular rate constants for two-water reaction were calculated using Eq.S3:

$$k(T, H_2O)_{\infty} = k_{H_2O}^{CVT}(T) \times K_{eq}(T, H_2O) \qquad \text{(3)}$$

The equilibrium constants $K_{eq}(T, H_2O)$ for the formation of complexes were calculated using Eq.S4 and EqS5:

$$K_{eq}^1(T, H_2O) = \frac{Q_{PRC-H_2O}}{Q_{CH_2NH-H_2O} \times Q_{H_2O}} \times \exp\left(-\frac{\Delta E_0^{(PRC-H_2O)_1}}{k_B T} \right). \qquad \text{(S4)}$$

$$K_{eq}^2(T, H_2O) = \frac{Q_{PRC-H_2O}}{Q_{H_2O-H_2O} \times Q_{CH_2NH}} \times \exp\left(-\frac{\Delta E_0^{(PRC-H_2O)_2}}{k_B T} \right). \qquad \text{(S5)}$$

where Q_{PRC-H_2O}, $Q_{CH_2NH-H_2O}$, $Q_{H_2O-H_2O}$ are total partition functions for different complexes, , and ΔE_0 is the zero-point corrected energies difference between pre-reactive complex and reactants.

The pressure-dependent bimolecular rate constants for CH_2NH +H_2O was calculated based on ref.

$$k_{tot}^{rec}(T, M) = K_{eq} k_{\infty}^{uni} f_{CH_2NH--H_2O)} \qquad \text{(S6)}$$

where $k_{¥}^{uni}(T)$ is the high-pressure limit rate constant for $CH_2NH \cdots H_2O \rightarrow CH_2NH + H_2O$, K_{eq} is the equilibrium constant and $f_{CH_2NH--H_2O}$ is the calculated fractional of $CH_2NH \cdots H_2O$ during simulations consisted of 10^6 stochastic trials

As shown in PES (Fig. 3), a kinetic scheme can be summarized as $CH_2NH + H_2O + H_2O$ <==> [CH_2NH---H_2O---H_2O] ==> Products And a rate constant (k_P) to form products can be computed as:

k_P=Keq x kf for tri-molecular reaction (See Figure S4)

or k_P=Keq x k_f x[H_2O] for pseudo bi-molecular reaction

where Keq is the thermal equilibrium constant for the $CH_2NH+H_2O+H_2O$ <==> [CH_2NH--H_2O--H_2O] that is calculated using THERMO code, and k_f is the rate constant for the [CH_2NH--H2O--H2O] ==> Products (from CVT/SCT), which is extremely slow and the rate-determining step. The K_{eq} is completely independent on a number of pathways (e.g. Pathways A and B) and how it can be reached. The equilibrium (K_{eq}) depends only on the starting-point and the end-point according to thermodynamic regulations.

Therefore, we believe, the kinetic scheme of $CH_2NH+H_2O+H_2O$ → CH_2NH---H_2O---H_2O →Products. The rate-determining step is at the step 2, which is the same in both pathways. Therefore, the correct equation to calculate the rate constant form reactants to products is expressed by Eq. 10

$$k_P = k_A^{eff} = k_B^{eff}$$

Scheme1: Reaction mechanism of methylenimine with water molecule to produce formaldehyde and ammonia *via* aminomethanol.

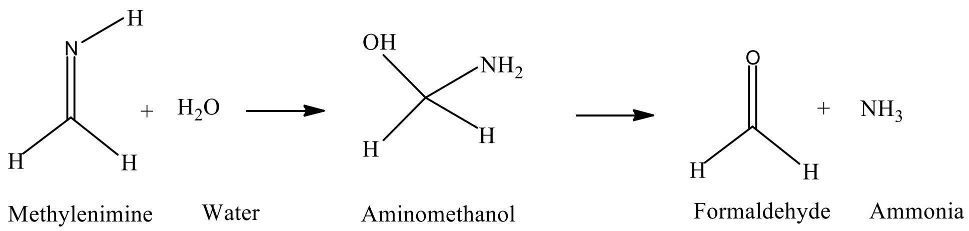

THERMO Input File

(1) $H_2O + H_2O \rightarrow H_2O{-}{-}{-}{-}H_2O$

```
KCAL  MCC
25
200. 225. 250. 275. 300. 325. 350. 375. 400. 500. 600. 700. 800. 900. 1000. 1100. 1200. 1300.
1400. 1500. 1600. 1700. 1800. 1900. 2000.
3

reac water 0.0 ! ATcT v.1.118a
H2O
! NIST-JANAF 1998
!
! small revisions based Bernath & Colin, JMS 257, 20-23 (2009)
2 1 1
0.0 1
5 'HAR' 'GHZ'
1 vib  1619.24      0  1
2 vib  3889.6       0  1
3 vib  3990.6       0  1
4 qro  830.14341    1  1 ! mo6
5 qro  350.9942744  1  2 ! mo6



reac water 0.0 ! ATcT v.1.118a
H2O
! NIST-JANAF 1998
!
! small revisions based Bernath & Colin, JMS 257, 20-23 (2009)
2 1 1
0.0 1
5 'HAR' 'GHZ'
1 vib  1619.24      0  1
2 vib  3889.6       0  1
3 vib  3990.6       0  1
4 qro  830.14341    1  1 ! mo6
5 qro  350.9942744  1  2 ! mo6

prod    H2O--H2O______    -2.9
H2OH2O
a) NIST / JANAF 1998
b) (blank comment line)
c) (blank comment line)
1    1    1
0.0 1
```

```
14      'HAR'   'GHZ'
1       vib     159.5   0       1
2       vib     172.65  0       1
3       vib     190.29  0       1
4       vib     207.41  0       1
5       vib     378.62  0       1
6       vib     635.48  0       1
7       vib     1622.83 0       1
8       vib     1638.71 0       1
9       vib     3799.08 0       1
10      vib     3885.07 0       1
11      vib     3965.18 0       1
12      vib     3982.55 0       1
13      qro     214.9604        1       1
14      qro     6.506562637     1       2
```

(2) $CH_2NH + H_2O \rightarrow CH_2NH\text{----}H_2O$

```
KCAL  MCC
25
200. 225. 250. 275. 300. 325. 350. 375. 400. 500. 600. 700. 800. 900. 1000. 1100. 1200. 1300.
1400. 1500. 1600. 1700. 1800. 1900. 2000.
3

reac     ch2nh_______   0.0
CH2NH
a) Frequencies from NIST Web-Book (11/2002)
b) Moments of Inertia from JANAF (1998)
c) delta-H(0 K) from JANAF (1998)
1  1  1
0.0     1
11    'HAR'     'GHZ'
1         vib       1076.990         1
2         vib       1122.380         1
3         vib       1169.680         1
4         vib       1370.410         1
5         vib       1501.130         1
6         vib       1744.990         1
7         vib       3068.770         1
8         vib       3153.020         1
9         vib       3471.720         1
10        qro       198.84379        1          1
11        qro       32.5443753       1          2



reac water 0.0 ! ATcT v.1.118a
H2O
! NIST-JANAF 1998
!
! small revisions based Bernath & Colin, JMS 257, 20-23 (2009)
```

```
2 1 1
0.0 1
5 'HAR' 'GHZ'
1 vib  1619.24      0  1
2 vib  3889.6       0  1
3 vib  3990.6       0  1
4 qro  830.14341    1  1 ! mo6
5 qro  350.9942744  1  2 ! mo6

prod    CH2NH--H2O______    -4.4
CH2NHH2O
a) NIST / JANAF 1998
b) (blank comment line)
c) (blank comment line)
1    1    1
0.0 1
20  'HAR'  'GHZ'
1       vib     118.71  0       1
2       vib     165.88  0       1
3       vib     208.14  0       1
4       vib     209.94  0       1
5       vib     371.3   0       1
6       vib     618.42  0       1
7       vib     1081.490        1
8       vib     1132.410        1
9       vib     1158.3  0       1
10      vib     1378.330        1
11      vib     1502.990        1
12      vib     1642.320        1
13      vib     1749.620        1
14      vib     3076.340        1
15      vib     3177.940        1
16      vib     3496.440        1
17      vib     3719.140        1
18      vib     3971.110        1
19      qro     34.27515        1       1
20      qro     4.696331507     1       2
```

(3) **$H_2O\cdots H_2O + CH_2NH \rightarrow$ PRC**

```
KCAL  MCC
25
200. 225. 250. 275. 300. 325. 350. 375. 400. 500. 600. 700. 800. 900. 1000. 1100.
1200. 1300. 1400. 1500. 1600. 1700. 1800. 1900. 2000.
3

reac    H2O--H2O______    0.0
H2OH2O
a) NIST / JANAF 1998
b) (blank comment line)
```

```
c) (blank comment line)
1    1    1
0.0 1
14        'HAR'  'GHZ'
1         vib       159.5   0          1
2         vib       172.65 0          1
3         vib       190.29 0          1
4         vib       207.41 0          1
5         vib       378.62 0          1
6         vib       635.48 0          1
7         vib       1622.83           0          1
8         vib       1638.71           0          1
9         vib       3799.08           0          1
10        vib       3885.07           0          1
11        vib       3965.18           0          1
12        vib       3982.55           0          1
13        qro       214.9604          1          1
14        qro       6.506562637   1          2
```

```
reac     ch2nh_______   0.0
CH2NH
a) Frequencies from NIST Web-Book (11/2002)
b) Moments of Inertia from JANAF (1998)
c) delta-H(0 K) from JANAF (1998)
1  1  1
0.0     1
11     'HAR'    'GHZ'
1      vib     1076.99 0       1
2      vib     1122.38 0       1
3      vib     1169.68 0       1
4      vib     1370.41 0       1
5      vib     1501.13 0       1
6      vib     1744.99 0       1
7      vib     3068.77 0       1
8      vib     3153.02 0       1
9      vib     3471.72 0       1
10      qro     198.84379       1       1
11      qro     32.5443753      1       2
```

```
prod    CH2NH--2H2O______    -8.60
CH2NHH4O2
a) NIST / JANAF 1998
```

b) (blank comment line)
c) (blank comment line)
1 1 1
0.0 1
29 'HAR' 'GHZ'

1	vib	64.38	0	1
2	vib	78.61	0	1
3	vib	164.37	0	1
4	vib	193.83	0	1
5	vib	211.46	0	1
6	vib	219.67	0	1
7	vib	233.19	0	1
8	vib	240.45	0	1
9	vib	412.69	0	1
10	vib	441.28	0	1
11	vib	704.08	0	1
12	vib	843.17	0	1
13	vib	1105.89	0	1
14	vib	1146.34	0	1
15	vib	1171.71	0	1
16	vib	1394.03	0	1
17	vib	1514.09	0	1
18	vib	1647.34	0	1
19	vib	1660.69	0	1
20	vib	1743.46	0	1
21	vib	3070.29	0	1
22	vib	3185.22	0	1
23	vib	3485.08	0	1
24	vib	3500.81	0	1
25	vib	3647.65	0	1
26	vib	3952.34	0	1
27	vib	3970.63	0	1
28	qro	6.13309	1	1
29	qro	2.697160156	1	2

(4) $CH_2NH\cdots H_2O$ + H2O→ PRC

KCAL MCC
25
200. 225. 250. 275. 300. 325. 350. 375. 400. 500. 600. 700. 800. 900. 1000. 1100. 1200. 1300. 1400. 1500. 1600. 1700. 1800. 1900. 2000.
3

reac CH2NH--H2O______ 0.0
CH2NHH2O
a) NIST / JANAF 1998
b) (blank comment line)
c) (blank comment line)
1 1 1
0.0 1
20 'HAR' 'GHZ'
1 vib 118.71 0 1
2 vib 165.88 0 1
3 vib 208.14 0 1
4 vib 209.94 0 1
5 vib 371.3 0 1
6 vib 618.42 0 1
7 vib 1081.49 0 1
8 vib 1132.41 0 1
9 vib 1158.3 0 1
10 vib 1378.33 0 1
11 vib 1502.99 0 1
12 vib 1642.32 0 1
13 vib 1749.62 0 1
14 vib 3076.34 0 1
15 vib 3177.94 0 1
16 vib 3496.44 0 1
17 vib 3719.14 0 1
18 vib 3971.11 0 1
19 qro 34.27515 1 1
20 qro 4.696331507 1 2

reac water 0.0 ! ATcT v.1.118a
H_2O
! NIST-JANAF 1998
!
! small revisions based Bernath & Colin, JMS 257, 20-23 (2009)
2 1 1
0.0 1
5 'HAR' 'GHZ'
1 vib 1619.24 0 1
2 vib 3889.6 0 1
3 vib 3990.6 0 1
4 qro 830.14341 1 1 ! mo6
5 qro 350.9942744 1 2 ! mo6

prod CH2NH--2H2O______ -7.2
CH2NHH4O2
a) NIST / JANAF 1998
b) (blank comment line)
c) (blank comment line)
1 1 1
0.0 1
29 'HAR' 'GHZ'

1	vib	64.38	0	1
2	vib	78.61	0	1
3	vib	164.37	0	1
4	vib	193.83	0	1
5	vib	211.46	0	1
6	vib	219.67	0	1
7	vib	233.19	0	1
8	vib	240.45	0	1
9	vib	412.69	0	1
10	vib	441.28	0	1
11	vib	704.08	0	1
12	vib	843.17	0	1
13	vib	1105.89	0	1
14	vib	1146.34	0	1
15	vib	1171.71	0	1
16	vib	1394.03	0	1
17	vib	1514.09	0	1
18	vib	1647.34	0	1
19	vib	1660.69	0	1
20	vib	1743.46	0	1
21	vib	3070.29	0	1
22	vib	3185.22	0	1
23	vib	3485.08	0	1
24	vib	3500.81	0	1
25	vib	3647.65	0	1
26	vib	3952.34	0	1
27	vib	3970.63	0	1
28	qro	6.13309	1	1
29	qro	2.697160156	1	2

KTOOLS Input File

```
CH2NH+H2O -> CH2O+NH3  ro-harmonic vib model M062X/pople  enthalpies

KCAL  MCC              ! Ekey, Sskey
both                   ! Whatdo
85000  10.             ! Emax Egrain
500    1               ! Jmax Jgrain
500  5000  85000.      ! 1D double array parameters: Imax1, Isize, Emax2
9                      ! Nt number of temperatures
200. 225. 250. 275. 300. 325. 350. 375.  400.
1 20   2               ! no. of reactants, no. of of trial transition states, no. of products

reac     CH2NH-H2O  0.0  0.0
CH2NHH2O
a) NIST / JANAF 2003, Dorofeeva et al. J. Phys. Chem. Ref. Data 32 (2003) 879
b) (blank comment line)
c) (blank comment line)
1   1   1
0.0  1
20     'HAR'   'GHZ'
1      vib    118.71  0      1
2      vib    165.88  0      1
3      vib    208.14  0      1
4      vib    209.94  0      1
5      vib    371.3   0      1
6      vib    618.42  0      1
7      vib    1081.49 0      1
8      vib    1132.41 0      1
9      vib    1158.3  0      1
10     vib    1378.33 0      1
11     vib    1502.99 0      1
12     vib    1642.32 0      1
13     vib    1749.62 0      1
14     vib    3076.34 0      1
15     vib    3177.94 0      1
16     vib    3496.44 0      1
17     vib    3719.14 0      1
18     vib    3971.11 0      1
19     kro    34.27515      1      1
20     jro    4.696331507    1      2


ctst     CH2NH-H2O-36       1.33    3.6      0
CH2NHH2O
a)       Akbar
b)       m062x
```

c) constrained optimization (OH-NO2 distance constrained)
1 1 1
0 1
19 'HAR' 'GHZ'
1 vib 59.79 0 1
2 vib 79.21 0 1
3 vib 188.08 0 1
4 vib 243.44 0 1
5 vib 467.44 0 1
6 vib 1087.09 0 1
7 vib 1121.84 0 1
8 vib 1166.9 0 1
9 vib 1310.87 0 1
10 vib 1495.28 0 1
11 vib 1630.43 0 1
12 vib 1727.08 0 1
13 vib 3062.84 0 1
14 vib 3158.59 0 1
15 vib 3469.53 0 1
16 vib 3794.74 0 1
17 vib 3957.12 0 1
18 kro 42.50561 1 1 ! GHz
19 jro 2.757894573 1 2 ! GHz

ctst CH2NH-H2O-38 1.95 3.8 0
CH2NHH2O
a) Akbar
b) m062x
c) constrained optimization (OH-NO2 distance constrained)
1 1 1
0 1
19 'HAR' 'GHZ'
1 vib 60.22 0 1
2 vib 67.41 0 1
3 vib 159.54 0 1
4 vib 223.58 0 1
5 vib 421.67 0 1
6 vib 1085.57 0 1
7 vib 1120.81 0 1
8 vib 1167.79 0 1
9 vib 1308.42 0 1
10 vib 1495.01 0 1
11 vib 1627.43 0 1
12 vib 1725.21 0 1
13 vib 3062.45 0 1

14 vib 3157.85 0 1
15 vib 3467.98 0 1
16 vib 3818.43 0 1
17 vib 3954.26 0 1
18 kro 43.1247 1 1 ! GHz
19 jro 2.499388937 1 2 ! GHz

ctst CH2NH-H2O-40 2.44 4 0
CH2NHH2O
a) Akbar
b) m062x
c) constrained optimization (ch2nh-h2o distance constrained)
1 1 1
0 1
19 'HAR' 'GHZ'
1 vib 57.28 0 1
2 vib 63.01 0 1
3 vib 126.47 0 1
4 vib 203.09 0 1
5 vib 377.94 0 1
6 vib 1084.44 0 1
7 vib 1120.54 0 1
8 vib 1168.69 0 1
9 vib 1306.69 0 1
10 vib 1494.86 0 1
11 vib 1625.39 0 1
12 vib 1723.79 0 1
13 vib 3063.15 0 1
14 vib 3158.01 0 1
15 vib 3464.99 0 1
16 vib 3835.43 0 1
17 vib 3952.58 0 1
18 kro 43.28132 1 1 ! GHz
19 jro 2.282428917 1 2 ! GHz

ctst CH2NH-H2O-42 2.99 4.2 0
CH2NHH2O
a) Nguyen, Barker,et al. (2011+)
b) UB97-1/m062x
c) constrained optimization (OH-NO2 distance constrained)
1 1 1
0 1
19 'HAR' 'GHZ'
1 vib 62.54 0 1

2 vib 77.09 0 1
3 vib 133.83 0 1
4 vib 208.64 0 1
5 vib 346.51 0 1
6 vib 1083.94 0 1
7 vib 1123.32 0 1
8 vib 1170.16 0 1
9 vib 1306.35 0 1
10 vib 1495.57 0 1
11 vib 1619.11 0 1
12 vib 1722.62 0 1
13 vib 3067.4 0 1
14 vib 3164.6 0 1
15 vib 3454.93 0 1
16 vib 3847.95 0 1
17 vib 3961.94 0 1
18 kro 43.29473 1 1 ! GHz
19 jro 2.093259836 1 2 ! GHz

ctst CH2NH-H2O-44 3.3 4.4 0
CH2NHH2O
a) Nguyen, Barker,et al. (2011+)
b) UB97-1/m062x
c) constrained optimization (OH-NO2 distance constrained)
1 1 1
0 1
19 'HAR' 'GHZ'
1 vib 60.07 0 1
2 vib 73.52 0 1
3 vib 142.06 0 1
4 vib 187.35 0 1
5 vib 302.04 0 1
6 vib 1083.37 0 1
7 vib 1122.64 0 1
8 vib 1170.57 0 1
9 vib 1305.7 0 1
10 vib 1495.47 0 1
11 vib 1618.4 0 1
12 vib 1722.53 0 1
13 vib 3066.82 0 1
14 vib 3163.48 0 1
15 vib 3453.84 0 1
16 vib 3848.22 0 1
17 vib 3965.5 0 1
18 kro 42.9707 1 1 ! GHz

19 jro 1.931292032 1 2 ! GHz

ctst CH2NH-H2O-46 3.61 4.6 0
CH2NHH2O
a) Nguyen, Barker,et al. (2011+)
b) UB97-1/m062x
c) constrained optimization (OH-NO2 distance constrained)
1 1 1
0 1
19 'HAR' 'GHZ'
1 vib 58.7 0 1
2 vib 74.1 0 1
3 vib 159.4 0 1
4 vib 183.08 0 1
5 vib 272.72 0 1
6 vib 1083.16 0 1
7 vib 1122.37 0 1
8 vib 1170.92 0 1
9 vib 1305.64 0 1
10 vib 1495.84 0 1
11 vib 1618.55 0 1
12 vib 1722.84 0 1
13 vib 3066.65 0 1
14 vib 3163.37 0 1
15 vib 3452.03 0 1
16 vib 3851.51 0 1
17 vib 3967.58 0 1
18 kro 42.64997 1 1 ! GHz
19 jro 1.786914092 1 2 ! GHz

ctst CH2NH-H2O-48 3.85 4.8 0
CH2NHH2O
a) Akbar
b) m062x
c) constrained optimization ch2nh-h2o distance constrained)
1 1 1
0 1
19 'HAR' 'GHZ'
1 vib 57.39 0 1
2 vib 79.4 0 1
3 vib 157.75 0 1
4 vib 182.17 0 1
5 vib 255.61 0 1
6 vib 1083.05 0 1
7 vib 1122.61 0 1
8 vib 1171.39 0 1

9 vib 1305.32 0 1
10 vib 1496.3 0 1
11 vib 1617.06 0 1
12 vib 1722.88 0 1
13 vib 3066.83 0 1
14 vib 3164.5 0 1
15 vib 3449.35 0 1
16 vib 3855.05 0 1
17 vib 3969.31 0 1
18 kro 42.40552 1 1 ! GHz
19 jro 1.657221088 1 2 ! GHz

ctst CH2NH-H2O-50 4.03 5 0
CH2NHH2O
a) Akbar
b) m062x
c) constrained optimization ch2nh-h2o distance constrained)
1 1 1
0 1
19 'HAR' 'GHZ'
1 vib 55.82 0 1
2 vib 81.76 0 1
3 vib 151.7 0 1
4 vib 181.85 0 1
5 vib 241.48 0 1
6 vib 1082.56 0 1
7 vib 1122.04 0 1
8 vib 1171.72 0 1
9 vib 1303.65 0 1
10 vib 1496.42 0 1
11 vib 1616.33 0 1
12 vib 1722.18 0 1
13 vib 3066.46 0 1
14 vib 3165.14 0 1
15 vib 3447.01 0 1
16 vib 3858.11 0 1
17 vib 3969.64 0 1
18 kro 42.16518 1 1 ! GHz
19 jro 1.541015543 1 2 ! GHz

ctst CH2NH-H2O-52 4.18 5.2 0
CH2NHH2O
a) Akbar
b) m062x
c) constrained optimization ch2nh-h2o distance constrained)
1 1 1

0 1
19 'HAR' 'GHZ'
1 vib 54.71 0 1
2 vib 83.13 0 1
3 vib 146.45 0 1
4 vib 180.68 0 1
5 vib 232.06 0 1
6 vib 1082.77 0 1
7 vib 1121.34 0 1
8 vib 1171.93 0 1
9 vib 1306.28 0 1
10 vib 1497.18 0 1
11 vib 1614.5 0 1
12 vib 1723.33 0 1
13 vib 3065.76 0 1
14 vib 3165.7 0 1
15 vib 3444.38 0 1
16 vib 3861.36 0 1
17 vib 3970.55 0 1
18 kro 41.9142 1 1 ! GHz
19 jro 1.436604909 1 2 ! GHz

ctst CH2NH-H2O-54 4.27 5.4 0
CH2NHH2O
a) Akbar
b) m062x
c) constrained optimization ch2nh-h2o distance constrained)
1 1 1
0 1
19 'HAR' 'GHZ'
1 vib 52.98 0 1
2 vib 83.8 0 1
3 vib 128.45 0 1
4 vib 177.82 0 1
5 vib 225.4 0 1
6 vib 1082.65 0 1
7 vib 1119.43 0 1
8 vib 1172.1 0 1
9 vib 1306.77 0 1
10 vib 1497.8 0 1
11 vib 1611.87 0 1
12 vib 1723.67 0 1
13 vib 3064.72 0 1
14 vib 3166.23 0 1
15 vib 3441.03 0 1

16 vib 3863.01 0 1
17 vib 3971.99 0 1
18 kro 41.64897 1 1 ! GHz
19 jro 1.342497332 1 2 ! GHz

ctst CH2NH-H2O-56 4.36 5.6 0
CH2NHH2O
a) Akbar
b) m062x
c) constrained optimization ch2nh-h2o distance constrained)
1 1 1
0 1
19 'HAR' 'GHZ'
1 vib 51.41 0 1
2 vib 84.18 0 1
3 vib 119.54 0 1
4 vib 175.65 0 1
5 vib 220.25 0 1
6 vib 1082.52 0 1
7 vib 1116.92 0 1
8 vib 1172.21 0 1
9 vib 1306.17 0 1
10 vib 1498.23 0 1
11 vib 1610.52 0 1
12 vib 1723.78 0 1
13 vib 3063.86 0 1
14 vib 3166.72 0 1
15 vib 3438.93 0 1
16 vib 3864.5 0 1
17 vib 3973.06 0 1
18 kro 41.48547 1 1 ! GHz
19 jro 1.256634808 1 2 ! GHz

ctst CH2NH-H2O-58 4.45 5.8 0
CH2NHH2O
a) Akbar
b) m062x
c) constrained optimization ch2nh-h2o distance constrained)
1 1 1
0 1
19 'HAR' 'GHZ'
1 vib 50.01 0 1
2 vib 84.24 0 1
3 vib 115.84 0 1
4 vib 174.45 0 1

5 vib 215.24 0 1
6 vib 1082.41 0 1
7 vib 1111.9 0 1
8 vib 1172.58 0 1
9 vib 1305.08 0 1
10 vib 1499.55 0 1
11 vib 1608.34 0 1
12 vib 1724.09 0 1
13 vib 3062.81 0 1
14 vib 3167.16 0 1
15 vib 3437.09 0 1
16 vib 3874.79 0 1
17 vib 3979.19 0 1
18 kro 41.33573 1 1 ! GHz
19 jro 1.178617706 1 2 ! GHz

ctst CH2NH-H2O-60 4.49 6 0
CH2NHH2O
a) Akbar
b) m062x
c) constrained optimization ch2nh-h2o distance constrained)
1 1 1
0 1
19 'HAR' 'GHZ'
1 vib 48.54 0 1
2 vib 82.61 0 1
3 vib 108.42 0 1
4 vib 173.36 0 1
5 vib 211.07 0 1
6 vib 1082.51 0 1
7 vib 1107.68 0 1
8 vib 1172.08 0 1
9 vib 1308.38 0 1
10 vib 1499.46 0 1
11 vib 1608.09 0 1
12 vib 1724.73 0 1
13 vib 3061.76 0 1
14 vib 3167.31 0 1
15 vib 3434.41 0 1
16 vib 3868.81 0 1
17 vib 3976.9 0 1
18 kro 41.1221 1 1 ! GHz
19 jro 1.107966872 1 2 ! GHz

ctst CH2NH-H2O-62 4.56 6.2 0
CH2NHH2O

a) Akbar
b) m062x
c) constrained optimization ch2nh-h2o distance constrained)
1 1 1
0 1
19 'HAR' 'GHZ'
1 vib 47.73 0 1
2 vib 81.65 0 1
3 vib 118.91 0 1
4 vib 174.43 0 1
5 vib 207.74 0 1
6 vib 1081.8 0 1
7 vib 1101.98 0 1
8 vib 1171.97 0 1
9 vib 1307.59 0 1
10 vib 1499.93 0 1
11 vib 1607.57 0 1
12 vib 1725.07 0 1
13 vib 3060.92 0 1
14 vib 3167.43 0 1
15 vib 3433.68 0 1
16 vib 3870.15 0 1
17 vib 3979.67 0 1
18 kro 40.99231 1 1 ! GHz
19 jro 1.043033628 1 2 ! GHz

ctst CH2NH-H2O-64 4.6 6.4 0
CH2NHH2O
a) Akbar
b) m062x
c) constrained optimization ch2nh-h2o distance constrained)
1 1 1
0 1
19 'HAR' 'GHZ'
1 vib 46.4 0 1
2 vib 79.96 0 1
3 vib 126.48 0 1
4 vib 175.1 0 1
5 vib 204.15 0 1
6 vib 1082.21 0 1
7 vib 1092.96 0 1
8 vib 1171.47 0 1
9 vib 1311.38 0 1
10 vib 1500.75 0 1
11 vib 1607.14 0 1

12 vib 1726.11 0 1
13 vib 3060.08 0 1
14 vib 3167.55 0 1
15 vib 3430.83 0 1
16 vib 3868.25 0 1
17 vib 3978.98 0 1
18 kro 40.82333 1 1 ! GHz
19 jro 0.983798054 1 2 ! GHz

ctst CH2NH-H2O-66 4.64 6.6 0
CH2NHH2O
a) Akbar
b) m062x
c) constrained optimization ch2nh-h2o distance constrained)
1 1 1
0 1
19 'HAR' 'GHZ'
1 vib 45.12 0 1
2 vib 78.33 0 1
3 vib 130.32 0 1
4 vib 174.49 0 1
5 vib 200.24 0 1
6 vib 1078.68 0 1
7 vib 1085.16 0 1
8 vib 1171.16 0 1
9 vib 1313.08 0 1
10 vib 1501.25 0 1
11 vib 1607.17 0 1
12 vib 1727.19 0 1
13 vib 3059.41 0 1
14 vib 3167.66 0 1
15 vib 3428.7 0 1
16 vib 3870.92 0 1
17 vib 3981.93 0 1
18 kro 40.68325 1 1 ! GHz
19 jro 0.983798054 1 2 ! GHz

ctst CH2NH-H2O-68 4.66 6.8 0
CH2NHH2O
a) Akbar
b) m062x
c) constrained optimization ch2nh-h2o distance constrained)
1 1 1
0 1
19 'HAR' 'GHZ'
1 vib 43.92 0 1

2 vib 76.95 0 1
3 vib 134.99 0 1
4 vib 172.67 0 1
5 vib 196.56 0 1
6 vib 1068.62 0 1
7 vib 1083.44 0 1
8 vib 1170.77 0 1
9 vib 1315.35 0 1
10 vib 1501.93 0 1
11 vib 1607.65 0 1
12 vib 1727.85 0 1
13 vib 3058.65 0 1
14 vib 3167.78 0 1
15 vib 3426.61 0 1
16 vib 3872.31 0 1
17 vib 3982.94 0 1
18 kro 40.56842 1 1 ! GHz
19 jro 0.879162502 1 2 ! GHz

ctst CH2NH-H2O-70 4.67 7.0 0
CH2NHH2O
a) Akbar
b) m062x
c) constrained optimization ch2nh-h2o distance constrained)
1 1 1
0 1
19 'HAR' 'GHZ'
1 vib 42.81 0 1
2 vib 75.29 0 1
3 vib 138.55 0 1
4 vib 168.53 0 1
5 vib 193.14 0 1
6 vib 1048.16 0 1
7 vib 1084.63 0 1
8 vib 1170.22 0 1
9 vib 1307.45 0 1
10 vib 1501.05 0 1
11 vib 1622.35 0 1
12 vib 1723.03 0 1
13 vib 3059 0 1
14 vib 3167.87 0 1
15 vib 3412.87 0 1
16 vib 3832.3 0 1
17 vib 3953.4 0 1
18 kro 40.45894 1 1 ! GHz
19 jro 0.832907923 1 2 ! GHz

ctst CH2NH-H2O-72 4.66 7.2 0
CH2NHH2O
a) Akbar
b) m062x
c) constrained optimization ch2nh-h2o distance constrained)
1 1 1
0 1
19 'HAR' 'GHZ'
1 vib 41.95 0 1
2 vib 73.7 0 1
3 vib 140.72 0 1
4 vib 159.33 0 1
5 vib 190.78 0 1
6 vib 1037.3 0 1
7 vib 1083.15 0 1
8 vib 1169.97 0 1
9 vib 1319.52 0 1
10 vib 1502.87 0 1
11 vib 1608.28 0 1
12 vib 1730.16 0 1
13 vib 3057.87 0 1
14 vib 3167.92 0 1
15 vib 3422.29 0 1
16 vib 3871.59 0 1
17 vib 3984.62 0 1
18 kro 40.35742 1 1 ! GHz
19 jro 0.790167478 1 2 ! GHz

ctst CH2NH-H2O-74 4.65 7.4 0
CH2NHH2O
a) Akbar
b) m062x
c) constrained optimization ch2nh-h2o distance constrained)
1 1 1
0 1
19 'HAR' 'GHZ'
1 vib 41.04 0 1
2 vib 72.01 0 1
3 vib 141.91 0 1
4 vib 144.47 0 1
5 vib 189.28 0 1
6 vib 1021.6 0 1
7 vib 1083.1 0 1
8 vib 1169.61 0 1
9 vib 1323.25 0 1

```
10      vib     1503.41      0     1
11      vib     1607.91      0     1
12      vib     1731.66      0     1
13      vib     3057.32      0     1
14      vib     3167.95      0     1
15      vib     3420.75      0     1
16      vib     3870.61      0     1
17      vib     3984.71      0     1
18      kro     40.2442      1     1     !     GHz
19      jro     0.7506513    1     2     !     GHz


prod ch2nh  4.4  0.0
CH2NH
a) Akbar
b) Calculated
c) from Akbar
1  1  1
0.0    1
11    'HAR'    'GHZ'
1      vib     1076.99 0      1
2      vib     1122.38 0      1
3      vib     1169.68 0      1
4      vib     1370.41 0      1
5      vib     1501.13 0      1
6      vib     1744.99 0      1
7      vib     3068.77 0      1
8      vib     3153.02 0      1
9      vib     3471.72 0      1
10      kro     198.84379      1      1
11      jro     32.5443753      1      2


prod  water  0.0  0.0
H2O
a) Akbar
b) Calculated
c) from Akbar
2 1 1
0.0 1
5 'HAR' 'GHZ'
1 vib  1619.24      0  1
2 vib  3889.6       0  1
3 vib  3990.6       0  1
4 kro  830.14341    1  1 ! mo6
```

5 jro 350.9942744 1 2 ! mo6
